# Correction: Changes in historical typhoid transmission across 16 U.S. cities, 1889–1931: Quantifying the impact of investments in water and sewer infrastructures

**DOI:** 10.1371/journal.pntd.0009347

**Published:** 2021-04-13

**Authors:** Maile T. Phillips, Katharine A. Owers, Bryan T. Grenfell, Virginia E. Pitzer

The images for Figs 1 and 2 are incorrectly switched. The image that appears as Fig 1 should be Fig 2, and the image that appears as Fig 2 should be Fig 1. The figure captions appear in the correct order. Please see the images in the correct order here.

**Fig 1:**
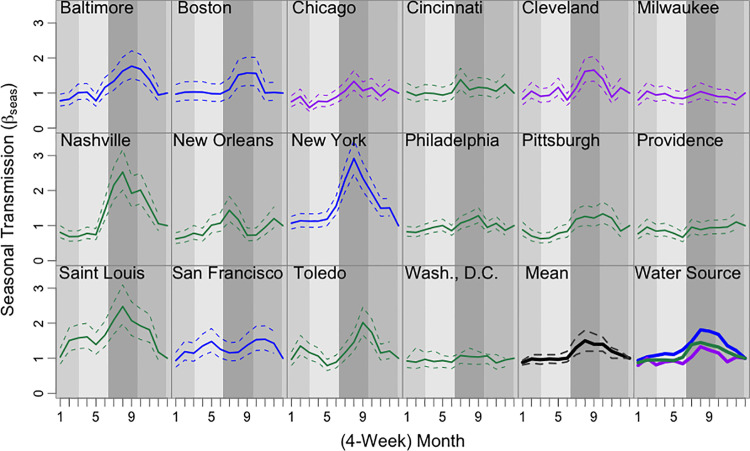
Annual seasonal typhoid transmission estimated from Time-series Susceptible-Infectious-Recovered models. The estimated seasonal transmission rate in each 4-week period is plotted for each city (color-coded by water source type; solid lines are the mean estimates and dashed lines are the 95% confidence intervals). The second-to-last panel shows the mean seasonal transmission across all cities in bold black. The last panel shows the mean seasonal transmission rate for cities with a particular water source type, with reservoirs in blue, rivers in green, and Great Lakes in purple. Seasons are shown in the background in shades of grey (medium-light grey for winter, light grey for spring, dark grey for summer, and medium-dark grey for fall).

**Fig 2:**
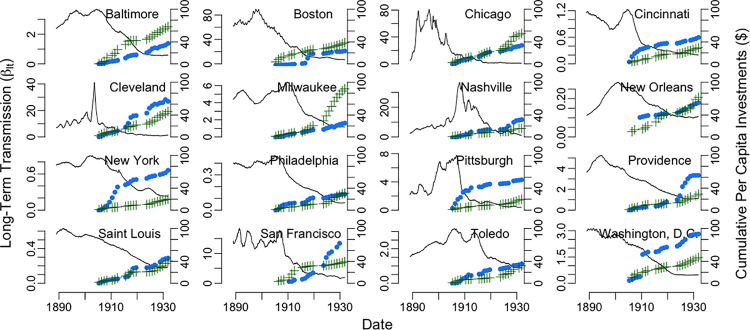
Long-term typhoid transmission rate by city estimated from Time-series Susceptible-Infectious-Recovered models. The estimated long-term transmission rate (β_lt_, solid black line) is plotted for each city, by four-week generation interval. Overall per capita investments in the water supply (blue circles) and sewer system (green pluses) in 1931 US dollars are also shown for each city from 1902–1931.
