# Supplementary material for: Changes in historical typhoid transmission across 16 U.S. cities, 1889-1931: Quantifying the impact of investments in water and sewer infrastructures
Source: PLoS Negl Trop Dis. 2020 Mar 18;14(3):e0008048. doi: 10.1371/journal.pntd.0008048 (PMC7105137; doi:10.1371/journal.pntd.0008048)
Supplement: S1 Text — (DOCX) [file pntd.0008048.s001.docx]

S1 Text: Model-fitting process.

We fit Time-series Susceptible-Infectious-Recovered (TSIR) models to each city’s time series to investigate seasonal and long-terms trends in typhoid transmission rates. In general, new infections at time *t*+1 (*I_t_*_+1_) arise from transmission from infectious (*I_t_*) to susceptible (*S_t_*) individuals at time *t*:

$I_{t+1}=\beta_{t}I_{t}^{\alpha}S_{t}$ *(S1)*

where $\beta_{t}$ is the disease transmission rate at time *t* and $\alpha$ is a scaling factor that adjusts for heterogeneous mixing in the population (*α*=1 corresponds to homogeneous mixing, *α*=0 corresponds to no auto-correlation in the time series).

After modifying Equation S1 to account for the unique features of typhoid epidemiology (Equation 2 main text) and log-transforming the model, the equation is as follows:

$log{(I}_{t+1})={log(\beta}_{lt})+log \left( \beta_{seas,j} \right)+\alpha log \left( I_{t}+C \right)+\log\left( S_{t} \right).$ *(S2)*

We reconstructed the infectious and chronic carrier populations via maximum likelihood estimation using Equation 5 (main text) and adjusting for underreporting. We then we modelled the susceptible population at time *t* as a function of the total population at time *t* minus the previously infectious and recovered individuals:

$S_{t}=N_{t}-\sum_{i=0}^{m} I_{t-i}\kappa_{i}$ (*S3*)

where $\kappa_{i}$is the degree of immunity *i* generation intervals after infection (determined by a decay of immunity function). We initially approximated *S_t_* using a log-transformation and first-degree Taylor series expansion around the average susceptible population size:

$\log\left( S_{t} \right)\approx\left[ \log\left( S_{mean} \right)+\frac{N_{t}}{S_{mean}}-1 \right]-\sum_{i=0}^{m} \frac{I_{t-i}\kappa_{i}}{S_{mean}}.$ (*S4*)

Penalized cubic splines on $\kappa$ were used to account for non-linearity in the duration and decay patterns in immunity. Equation S2 can then be rewritten as:

$log{(I}_{t+1})={log(\beta}_{lt})+log \left( \beta_{seas,j} \right)+\alpha log \left( I_{t}+C \right)$

$-\sum_{i=0}^{m} \frac{I_{t-i}\kappa_{i}}{S_{mean}}+\left[ \log\left( S_{mean} \right)+\frac{N_{t}}{S_{mean}}-1 \right]$ *(S5)*

Note that the waning of immunity affects the number of susceptible individuals at time *t*, thereby indirectly affecting the number of new infections at time *t+1*. Since the duration of immunity to typhoid infection and disease is not well understood, we performed sensitivity analyses exploring different durations of immunity (S2 Text).

We used Equation S5 and the semi-parametric method described by Koelle and Pascual (1, 2) to estimate the variation in $\beta_{lt}$ over the full 43-year study period from 1889-1931. One value of $\beta_{lt}$ was estimated for each four-week period from 1889-1931 (except for the first, to be able to calculate $I_{t+1}$), resulting in 558 estimations. We also estimated 13 values of $\beta_{seas,j}$ corresponding to transmission during the same four-week period each year. This parameter was estimated as a categorical variable with 13 values, where the first 12 four-week months were estimated in comparison to the last month ($\beta_{seas,13}=1)$.

To fit the model, we used a back-fitting algorithm comprised of repeated penalized cubic splines on $\kappa$, recursive first-order Taylor series expansion approximations on the $log(S_{t})$ term, and weighted least squares regressions on iterations of Equation S5. The iterative process was necessary to allow for the more accurate approximation of the Taylor series expansion and the cubic splines to converge. For the weighted least squares regressions, the weights were calculated as **I-W**, where **I** was the identity matrix of the same dimension, and **W** was the truncated Gaussian kernel weight matrix calculated from the spline penalty weights.

After the above algorithm converged, we used the same truncated Gaussian kernel weight matrix **W** to smooth the residuals of the model fit with all other parameters estimated. The smoothed residuals were then used to estimate the nonparametric variation in the long-term transmission rate ($\beta_{lt}$). The final long-term transmission rate was multiplied by the population at each time point to calculate per capita estimates.

The smoothing parameter and spline penalty weights were selected using cross-validation and testing across a range of values. Each city was fit using all possible values of the smoothing parameter from 1 to 35 in one-unit increments and spline penalty values from -2 to 30 in one-unit increments. If the parameters chosen were at the ends of the respective ranges, the intervals were extended until the optimal values fell within the values tested. To identify the optimal model, we used leave-one-out validation, in which we dropped one data point at a time (for all data points), fit the model to the remaining data, and then used the fitted model to predict the out-of-sample data point. We calculated the sum of squared differences between each point and its out-of-sample prediction over all points, and stored this as the cross-validated (CV) value for each model fit. The optimal model for each city was the one with the smallest CV value.

REFERENCES

1. Koelle K, Pascual M. Disentangling extrinsic from intrinsic factors in disease dynamics: a nonlinear time series approach with an application to cholera. The American naturalist. 2004;163(6):901-13, DOI: 10.1086/420798.

2. Koelle K, Rodó X, Pascual M, Yunus M, Mostafa G. Refractory periods and climate forcing in cholera dynamics. Nature. 2005;436(7051):696, DOI: 10.1038/nature03820.
