# Supplementary material for: Changes in historical typhoid transmission across 16 U.S. cities, 1889-1931: Quantifying the impact of investments in water and sewer infrastructures
Source: PLoS Negl Trop Dis. 2020 Mar 18;14(3):e0008048. doi: 10.1371/journal.pntd.0008048 (PMC7105137; doi:10.1371/journal.pntd.0008048)
Supplement: S2 Text — (DOCX) [file pntd.0008048.s002.docx]

S2 Text: Sensitivity analyses.

**Description of different components of the model that were tested**

We performed several sensitivity analyses to test the robustness of our model assumptions. We examined different components of the model, including our method of imputing missing data, the addition of small values to the reported death counts prior to log-transformation (to avoid taking the log of zero), the duration (or inclusion) of waning immunity, and the inclusion of chronic carriers in our TSIR models. We determined the impact of these assumptions on the seasonal and long-term transmission parameters, the relationship with the overall investment variables identified by the hierarchical regression models, and estimates of the heterogeneous mixing parameter (*α*). The results of these sensitivity analyses can be found in S21-36 Figs and S8-10 tables.

***Missing data***

To address the issue of missing data, we had to make some decisions about what the meaning of the missing values might be. In many instances, cities only reported typhoid deaths if there were any; however, we had to differentiate between these zero counts and truly missing data points. For the primary analysis, we used a cut-off of 13 consecutive weeks to differentiate between when missing values represented zeros versus missing data. If there were fewer than 13 weeks of missing death counts, the data points were coded as zeroes, and if there were more, we used Kalman smoothing to impute the missing values based on the previous observation and the “filter,” updated at each time point (1, 2). Since this 13-week cut-off was arbitrarily chosen, we also fit the TSIR models using an 8-week and 26-week cut-off and compared the results.

***Log-transformation***

Some of the death counts were zero, which posed a problem for the logarithmic transformation in our main TSIR model equation. In our main analysis, we added one to every data point to preserve the shape of the distribution. As a sensitivity analysis, we re-ran all of the models instead adding 0.5 to every data point to compare the impact of this assumption.

***Duration of immunity***

Waning of immunity against typhoid infection is poorly understood. While epidemiological studies and human challenge studies indicate that individuals can be re-infected with typhoid after approximately one year, mathematical models of typhoid infection consistently estimate that immunity to typhoid disease is long-lived in order to explain why the incidence tends to decline with age, particularly in high incidence settings. We assessed the assumption of waning immunity in our model, fitting additional models assuming only one year of immunity following infection and models with no waning of immunity (i.e. lifelong immunity following infection).

***Chronic carriers***

In fitting the TSIR models, we noted that the heterogeneous mixing parameter ($\alpha)$ was lower than estimated for other pathogens (e.g. measles) using TSIR models (3). We hypothesized that this was likely due to the contribution of long-cycle transmission in the epidemiology of typhoid, i.e. transmission from chronic carriers and the environmental reservoir. To test this, we compared the *α* values estimated for TSIR models without waning immunity, without chronic carriers, or without either, and in the simple TSIR model (Equation S1).

**Results of sensitivity analyses**

The results of the analyses in this study were generally robust to the changes in the assumptions examined. The seasonal transmission rates remained almost unchanged, regardless of variations in missing data imputation, addition of small amounts to the reported death counts, and duration or exclusion of waning immunity (S21-36 Figs, top half of panels).

The long-term transmission rates mostly retained their overall shape, but the scale of the transmission rate changed in some instances (S21-36 Figs, bottom half of the panels). However, the results of the hierarchical regression models with the overall financial variables were all quite similar, and results were mostly within 2% of the original estimates (S8-9 Tables).

The heterogeneous mixing parameter varied slightly between the different models, but mostly kept their order between cities (S10 Table). New Orleans, New York, Philadelphia, and Pittsburgh typically had the highest estimated *α* values regardless of the model formulation. The highest *α* values were estimated for the model with no immunity, suggesting that there may be some identifiability issues between the duration of immunity and the heterogeneous mixing parameter. Nevertheless, our results were robust to different durations of immunity, as noted above. Removing chronic carriers from the model generally led to estimated *α* values closer to zero, as expected, suggesting typhoid incidence is less dependent on acute cases in the previous generation. This suggests that long-cycle transmission of typhoid, which does not occur in direct proportion to cases in the previous generation, can help to explain some of the lack of autocorrelation in the data. Long-cycle transmission may be related to cases occurring two to three generations prior (since evidence suggests typhoid bacteria is not long-lived in the environment (4)) and/or cases residing in surrounding populations that could contaminate water catchment areas, which would not be captured by our model.

REFERENCES

1. Chui CK, Chen G. Kalman Filtering with Real-Time Applications. 3 ed. Huang TS, Kohonen T, Schroeder MR, editors. Berlin: Springer-Verlag; 1999.

2. Harvey AC. Forecasting, structural times series models and the Kalman filter. Cambridge, UK: Press Syndicate of the University of Cambridge; 1989.

3. Bjørnstad O, Finkenstädt B, Grenfell B. Dynamics of measles epidemics: Estimating scaling of transmission rates using a Time Series SIR model. Ecological Monographs. 2002;72(2), DOI: 10.1890/0012-9615(2002)072[0169:DOMEES]2.0.CO;2.

4. Cho J-C, Kim S-J. Viable, but non-culturable, state of a green fluorescence protein-tagged environmental isolate of Salmonella typhi in groundwater and pond water. FEMS Microbiology Letters. 1999;170(1):257-64, DOI: 10.1111/j.1574-6968.1999.tb13382.x.
