# Supplementary figures and images for: Changes in historical typhoid transmission across 16 U.S. cities, 1889-1931: Quantifying the impact of investments in water and sewer infrastructures
Source: PLoS Negl Trop Dis. 2020 Mar 18;14(3):e0008048. doi: 10.1371/journal.pntd.0008048 (PMC7105137; doi:10.1371/journal.pntd.0008048)

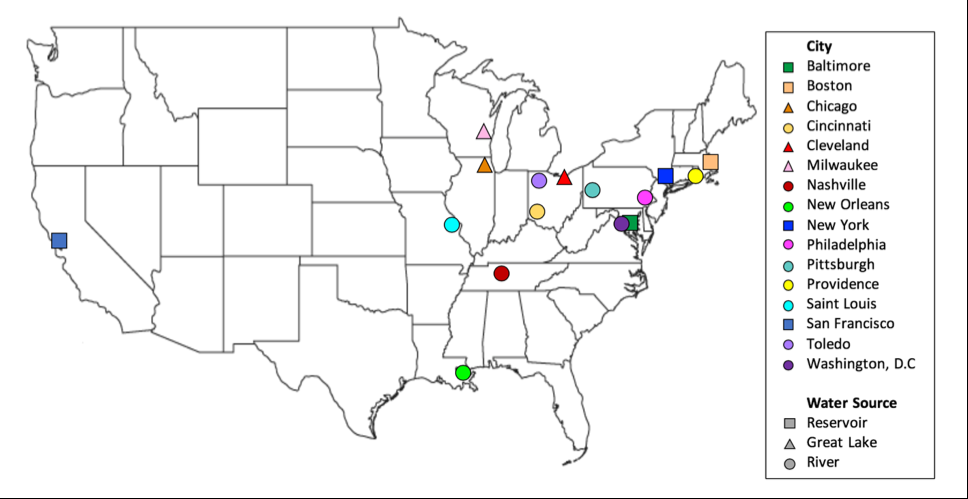

Supplement: S1 Fig — Each city included in the analysis is denoted by a different color in its geographical location in the United States. Squares denote cities with reservoirs, triangles denote those using the Great Lakes, and circles denote those with rivers as their main water source. The underlying map is adapted from the United States Geological Survey LandsatLook < https://landlook.usgs.gov/viewer.html#>. (TIF) [file pntd.0008048.s003.tif]

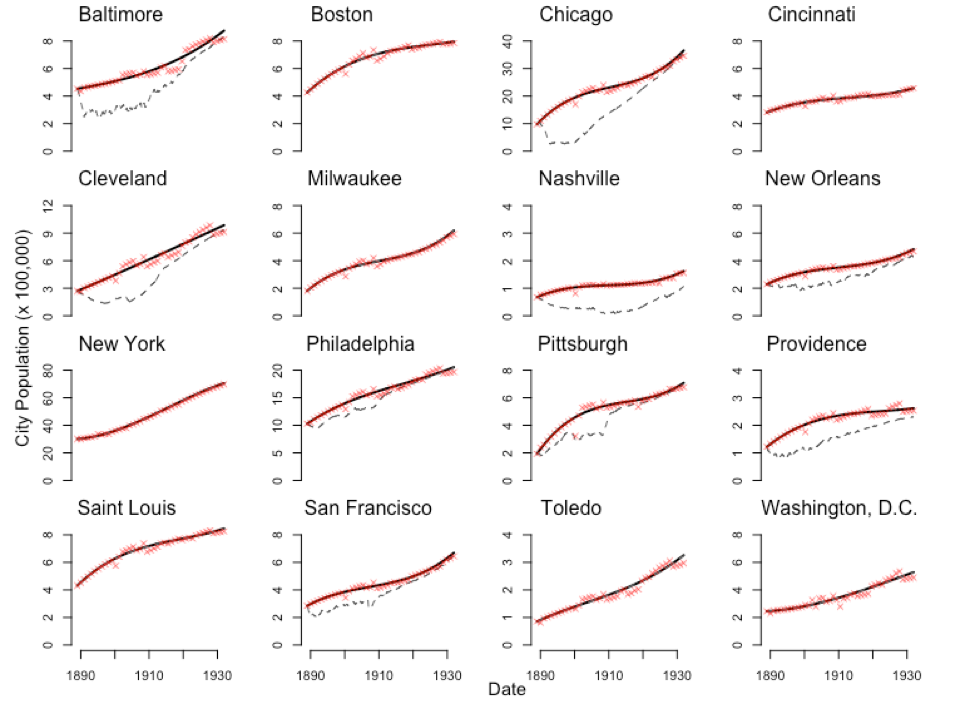

Supplement: S2 Fig — The yearly U.S. Census Bureau reported population (red Xs), monthly population extrapolated using cubic splines (solid black line), and susceptible population (dashed black line) estimated from the main TSIR models are shown for each city over the study period. Note that in some cities, the susceptible and total population are very close and cannot be differentiated in the plots. (TIF) [file pntd.0008048.s004.tif]

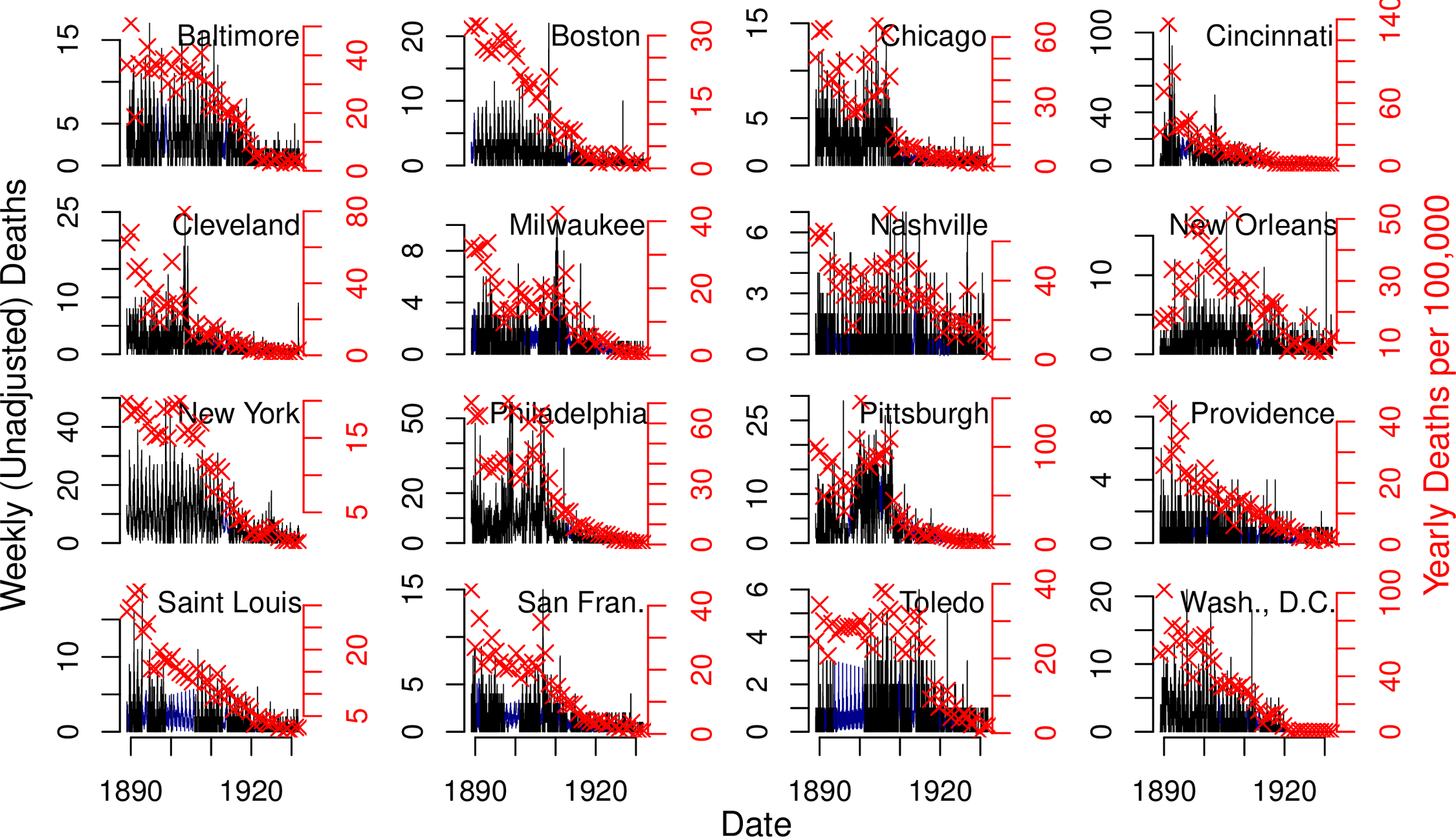

Supplement: S3 Fig — The observed (including imputation, in blue) time series of weekly deaths reportedly due to typhoid (black lines) and the yearly typhoid deaths per 100,000 people (red Xs) is shown for each city from 1889–1931. (TIF) [file pntd.0008048.s005.tif]

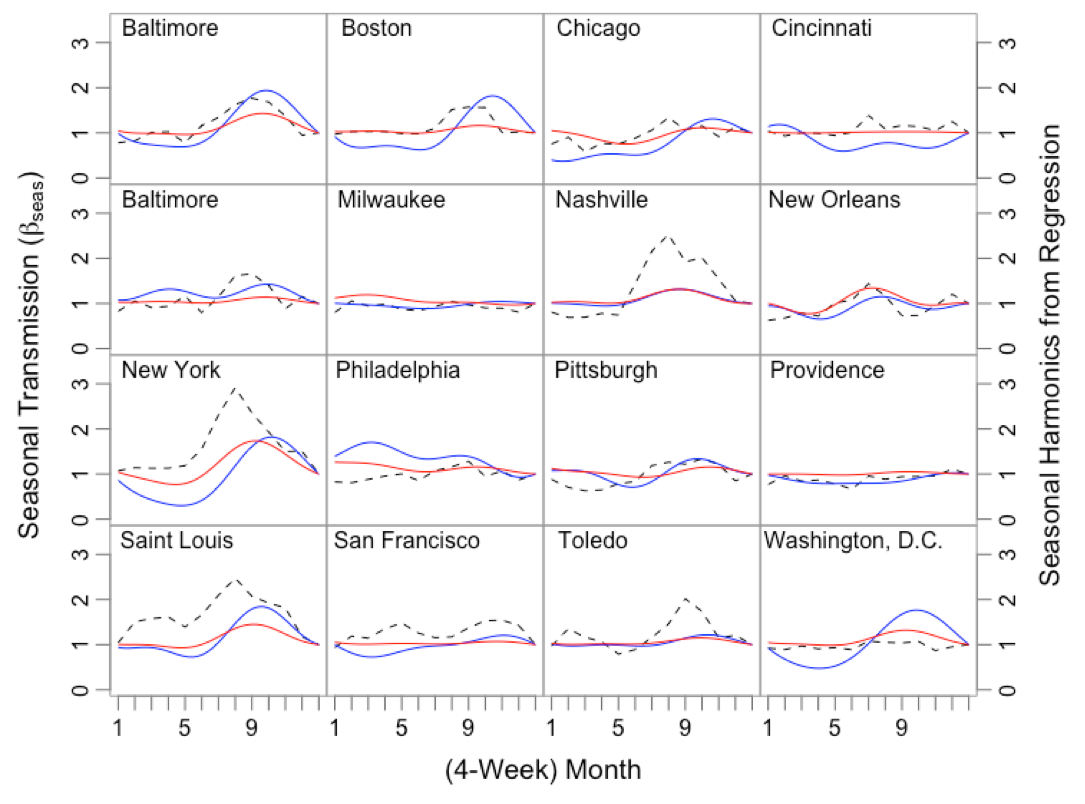

Supplement: S4 Fig — The pre- (blue) and post-intervention (red) six- and 12-month sinusoid curves fitted to the typhoid mortality data are shown for each city, along with the seasonal transmission rate estimated by the main TSIR model (dashed black line). (TIF) [file pntd.0008048.s006.tif]

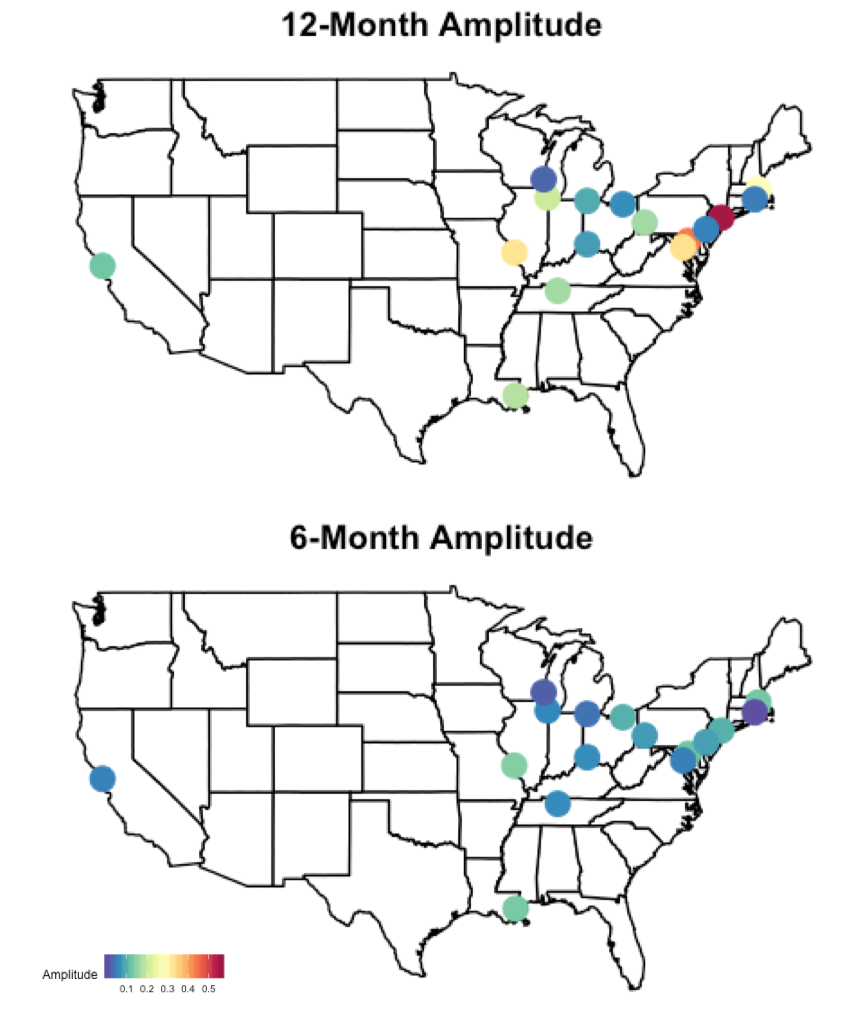

Supplement: S5 Fig — The average 12- and 6-month amplitudes of seasonal variation in reported typhoid mortality estimated from the harmonic regression analyses are shown separately according to the color scale and plotted by geographic location. (TIF) [file pntd.0008048.s007.tif]

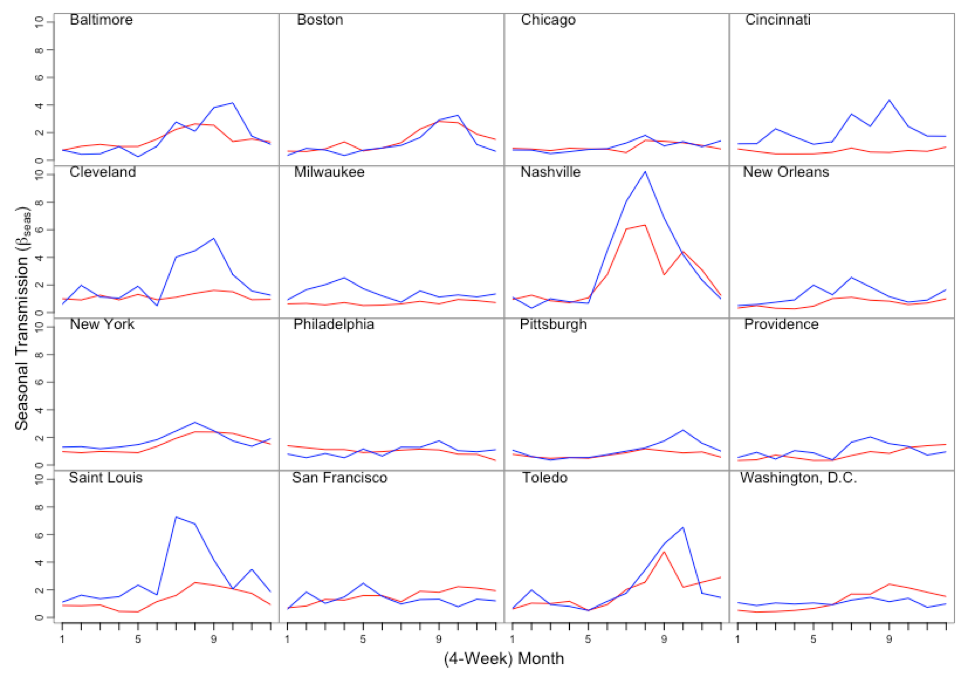

Supplement: S6 Fig — The estimated four-week seasonal transmission rates extracted from each city’s simple TSIR model (not including waning of immunity) are shown for each pre- (blue) and post- (red) water supply intervention period. (TIF) [file pntd.0008048.s008.tif]

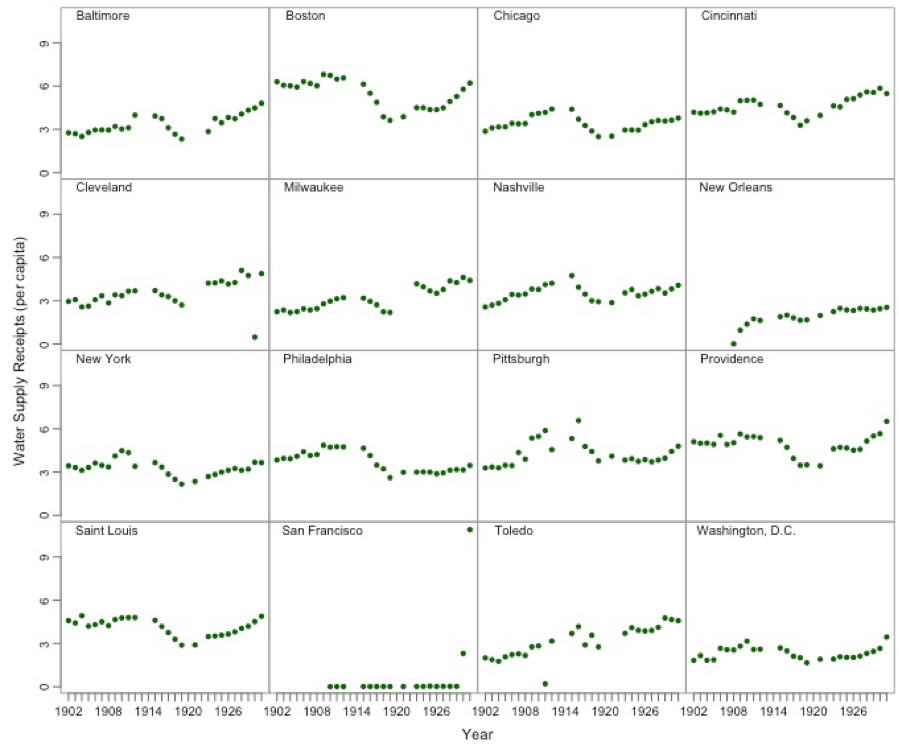

Supplement: S7 Fig — Annual water supply receipts from 1902–1931 are shown for each city in per capita increments (US$ per person). Dollar amounts are adjusted for inflation to 1931 US$. (TIF) [file pntd.0008048.s009.tif]

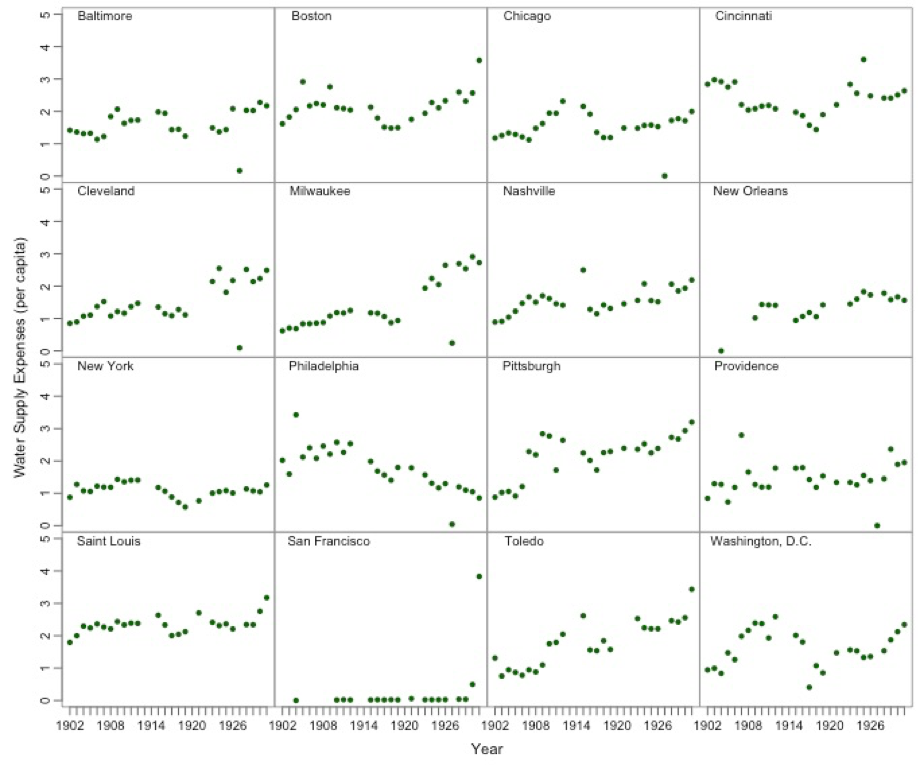

Supplement: S8 Fig — Annual spending on water supply expenses from 1902–1931 is shown for each city in per capita increments (US$ per person). Dollar amounts are adjusted for inflation to 1931 US$. (TIF) [file pntd.0008048.s010.tif]

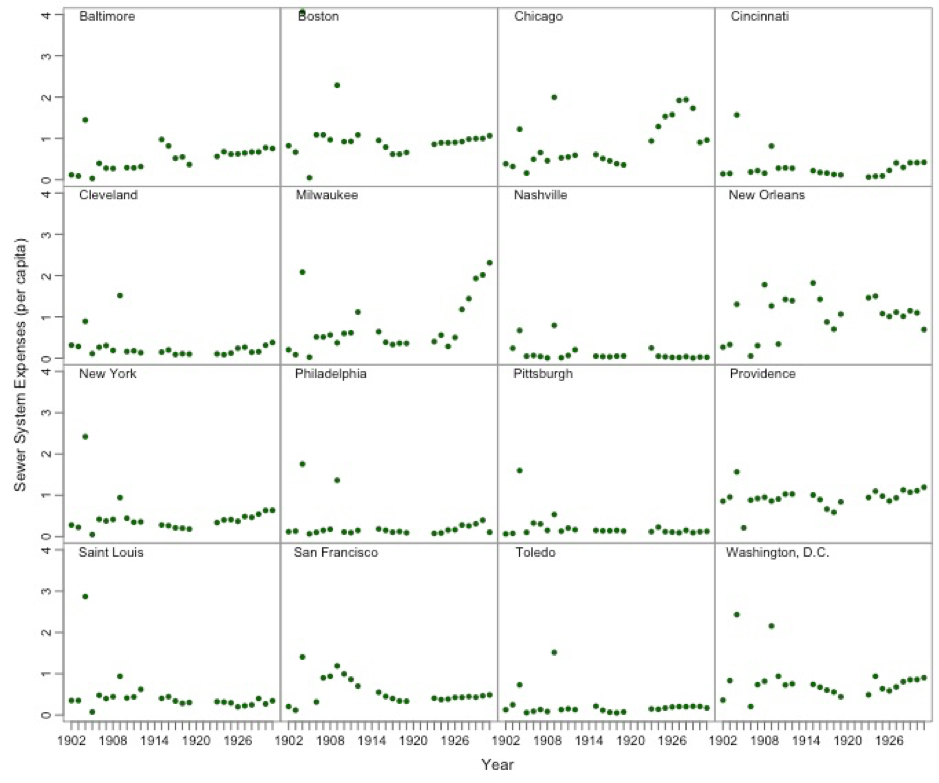

Supplement: S9 Fig — Annual spending on sewer system expenses from 1902–1931 is shown for each city in per capita increments (US$ per person). Dollar amounts are adjusted for inflation to 1931 US$. (TIF) [file pntd.0008048.s011.tif]

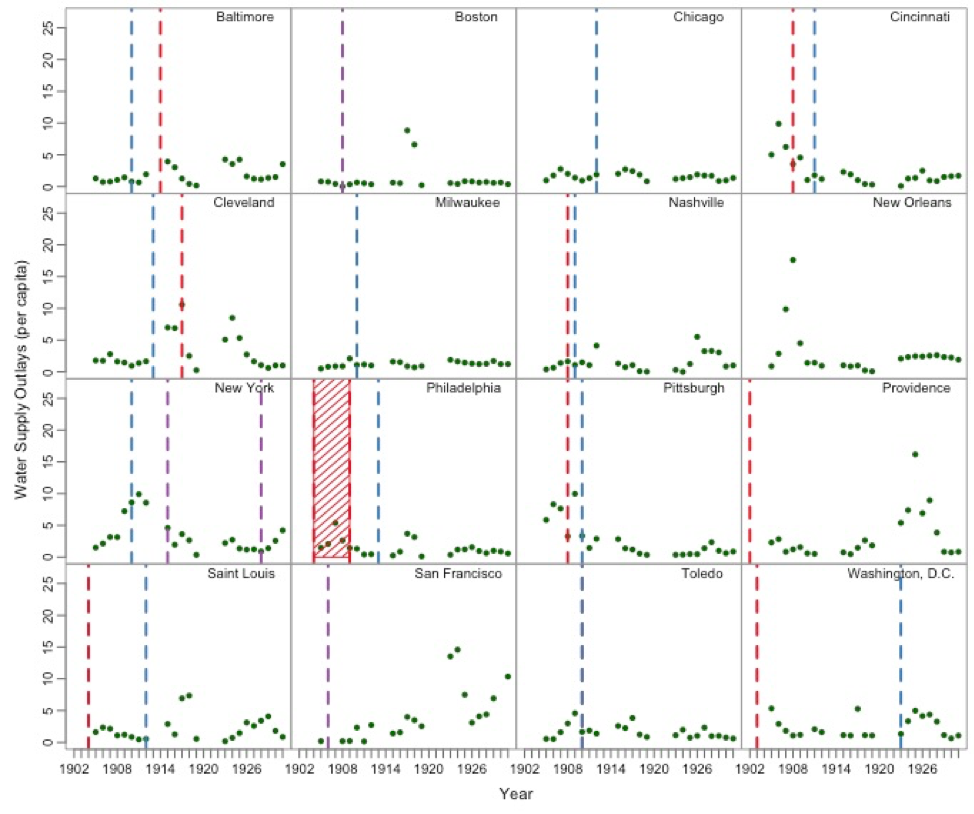

Supplement: S10 Fig — Annual spending on water supply outlays from 1902–1931 is shown for each city (green dots) in per capita increments (US$ per person). The year in which interventions were introduced are represented by the dashed lines for filtration (red), chlorination (blue), or other interventions (purple). The inclusion of intervention dates is for illustrative purposes. Outliers not seen: In 1930, water supply outlays from San Francisco totalled $70.97 per capita; this was the year in which the city purchased the water supply previously owned and operated by the Spring Valley Water Company. Chicago and New Orleans introduced water supply interventions in 1900, prior to the time period shown. Dollar amounts are adjusted for inflation to 1931 US$. (TIF) [file pntd.0008048.s012.tif]

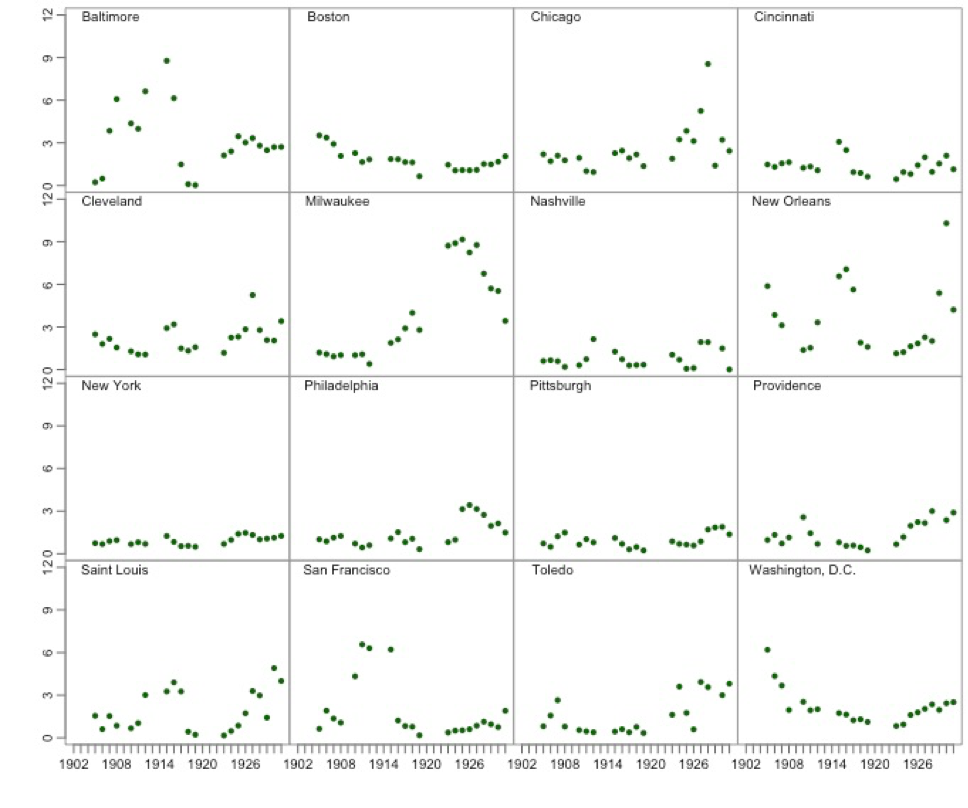

Supplement: S11 Fig — Annual spending on sewer system outlays from 1902–1931 is shown for each city in per capita increments (US$ per person). Dollar amounts are adjusted for inflation to 1931 US$. (TIF) [file pntd.0008048.s013.tif]

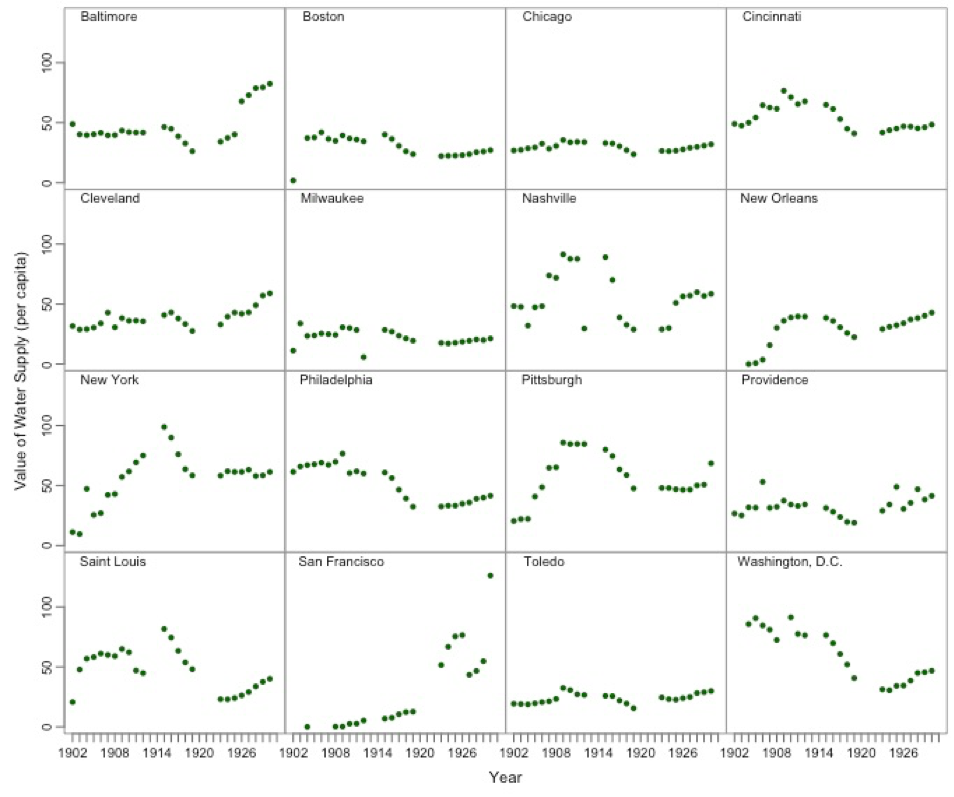

Supplement: S12 Fig — The overall annual value of the water supply system from 1902–1931 is shown for each city in per capita increments (US$ per person). Outliers not seen: In 1897, the value of the water supply system totalled $508.55 per capita in Washington, D.C. Dollar amounts are adjusted for inflation to 1931 US$. (TIF) [file pntd.0008048.s014.tif]

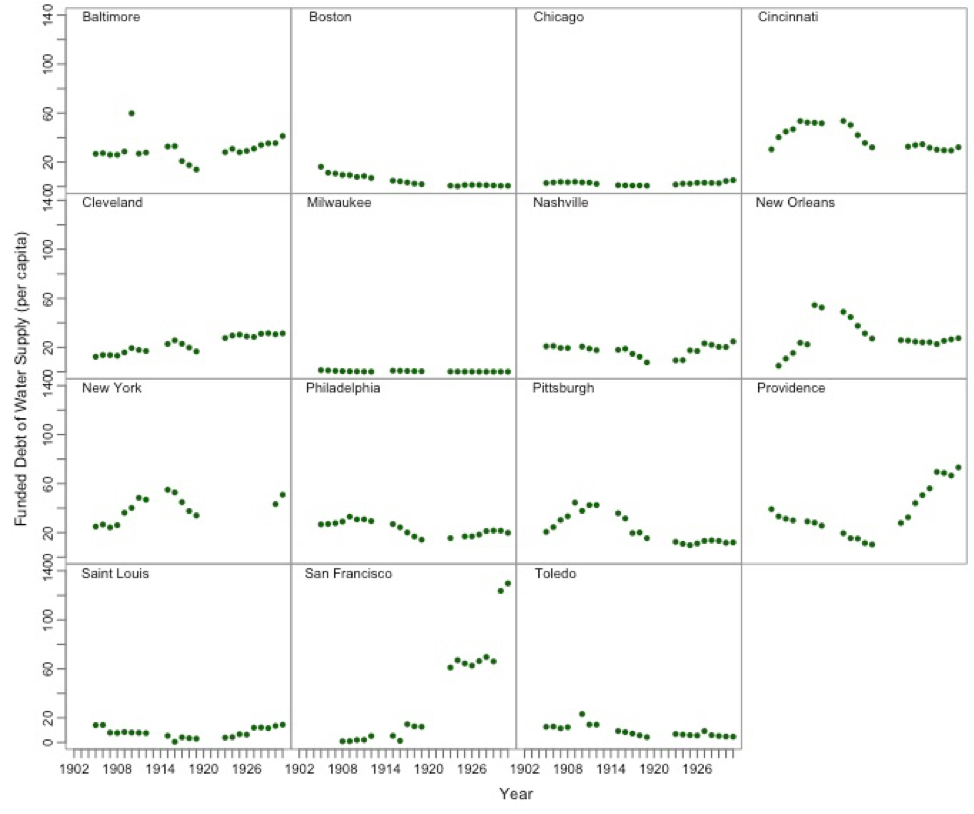

Supplement: S13 Fig — The overall annual accrued debt and/or funded loans for the water supply system from 1902–1931 is shown for each city in per capita increments (US$ per person). Note: Data were not available for this variable in Washington, D.C. Dollar amounts are adjusted for inflation to 1931 US$. (TIF) [file pntd.0008048.s015.tif]

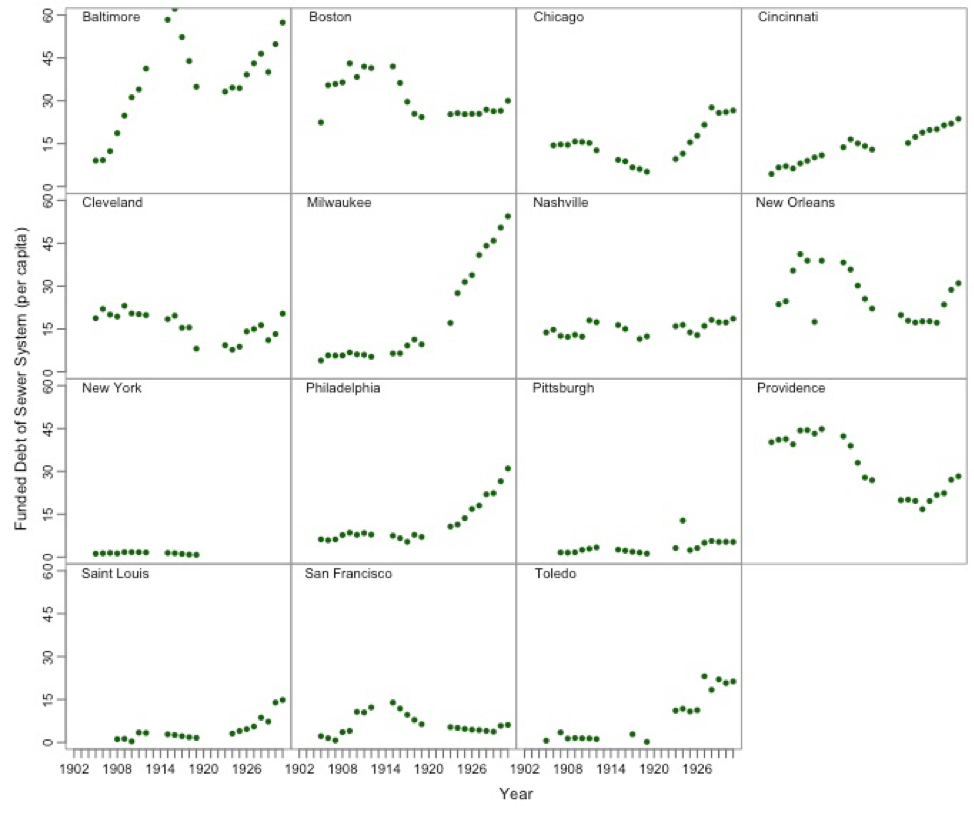

Supplement: S14 Fig — The overall annual accrued debt and/or funded loans for the sewer system from 1902–1931 are shown for each city in per capita increments (US$ per person). Note: Data were not available for this variable in Washington, D.C. Dollar amounts are adjusted for inflation to 1931 US$. (TIF) [file pntd.0008048.s016.tif]

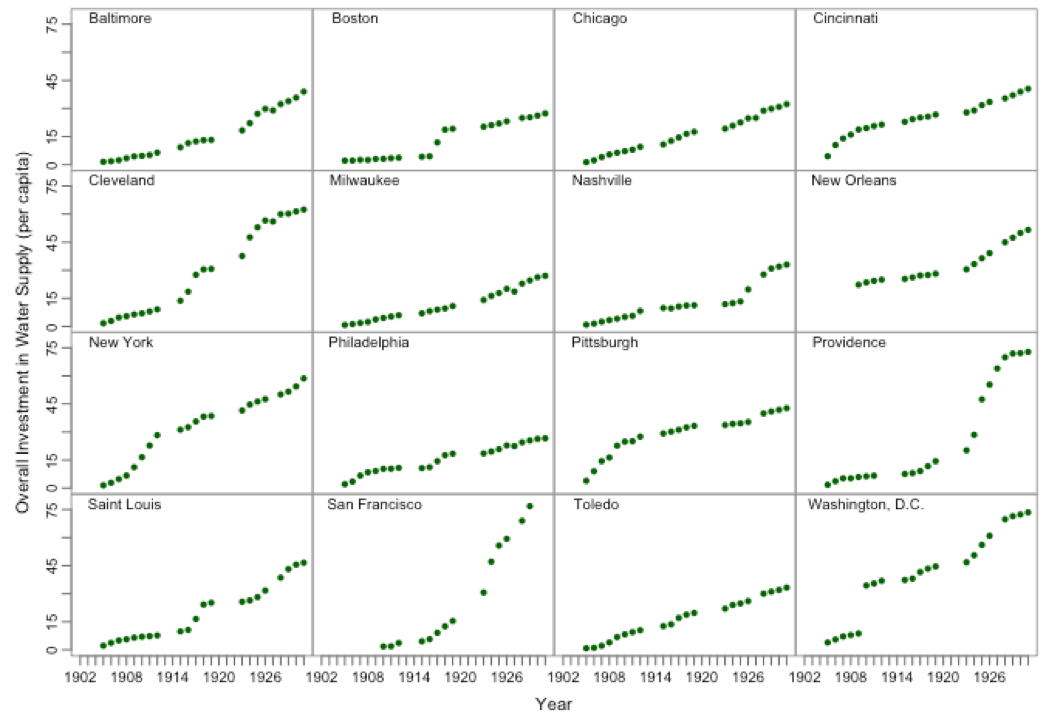

Supplement: S15 Fig — The overall cumulative investments in the water supply system from 1902–1931 are shown for each city in per capita increments (US$ per person). This was calculated as the cumulative sum of annual expenses and annual outlays minus annual receipts for the water supply system. Dollar amounts are adjusted for inflation to 1931 US$. (TIF) [file pntd.0008048.s017.tif]

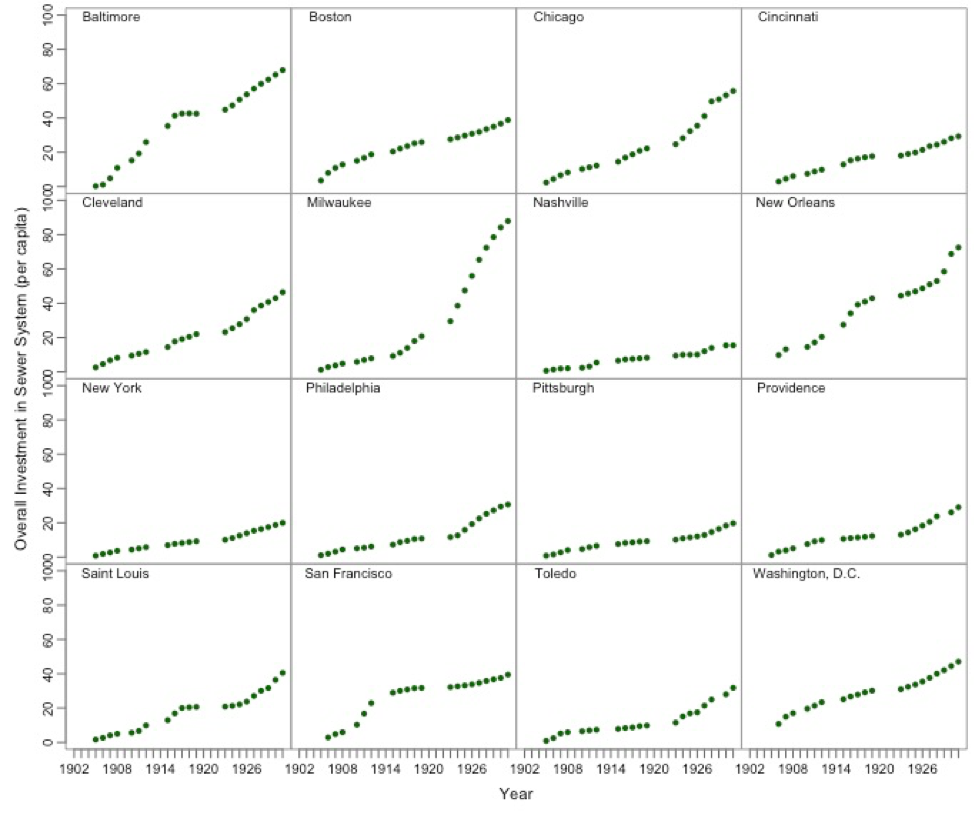

Supplement: S16 Fig — The overall cumulative investments in the sewer system from 1902–1931 are shown for each city in per capita increments (US$ per person). This was calculated as the cumulative sum of annual expenses and annual outlays for the sewer system. Dollar amounts are adjusted for inflation to 1931 US$. (TIF) [file pntd.0008048.s018.tif]

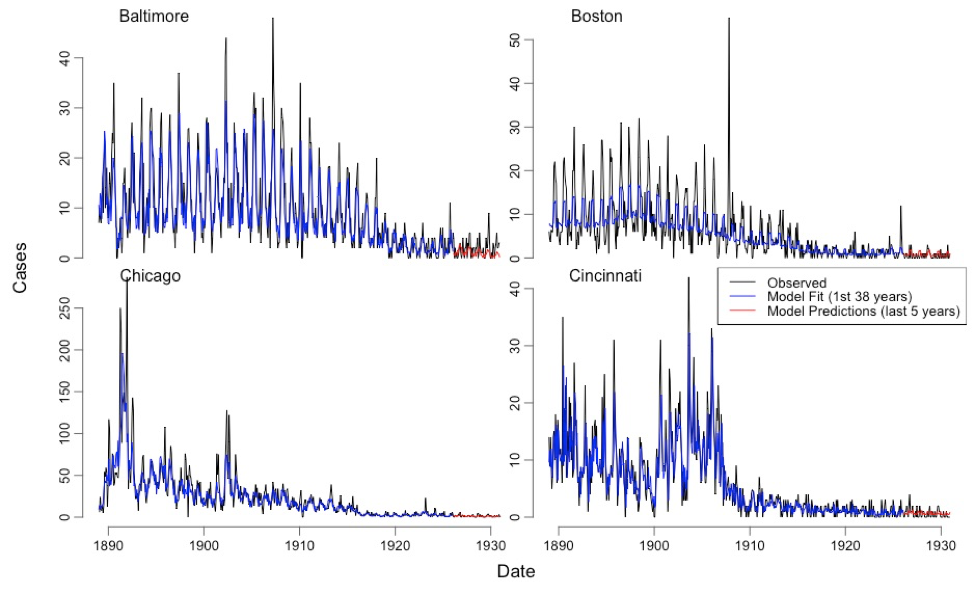

Supplement: S17 Fig — For each city, the TSIR model is fit using the first 38 years of data, then used to predict the last 5 years of data. In each plot, the observed data is shown in black, the model fit to the first 38 years is shown in blue, and the predicted last 5 years is shown in red. (TIF) [file pntd.0008048.s019.tif]

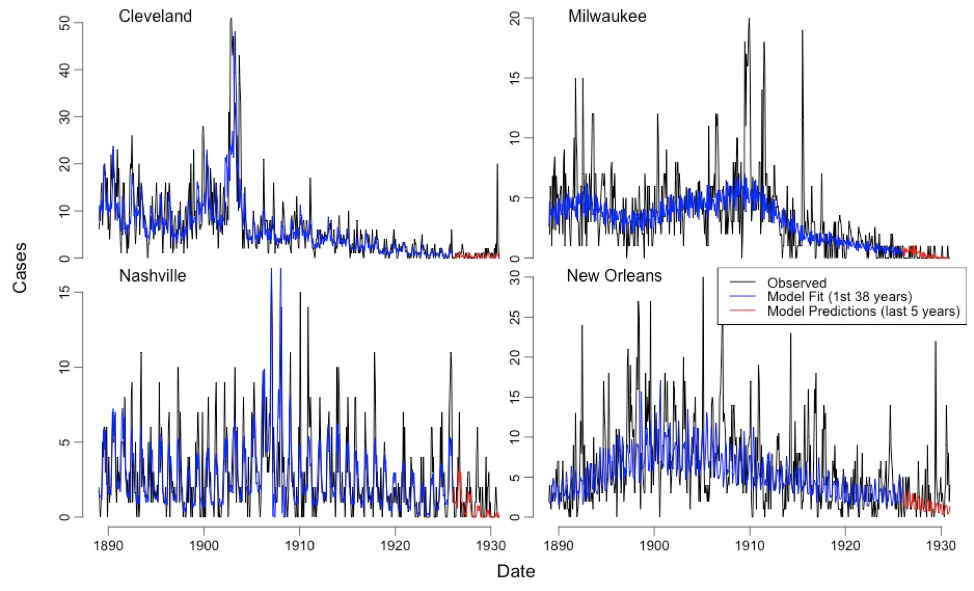

Supplement: S18 Fig — For each city, the TSIR model is fit using the first 38 years of data, then used to predict the last 5 years of data. In each plot, the observed data is shown in black, the model fit to the first 38 years is shown in blue, and the predicted last 5 years is shown in red. (TIF) [file pntd.0008048.s020.tif]

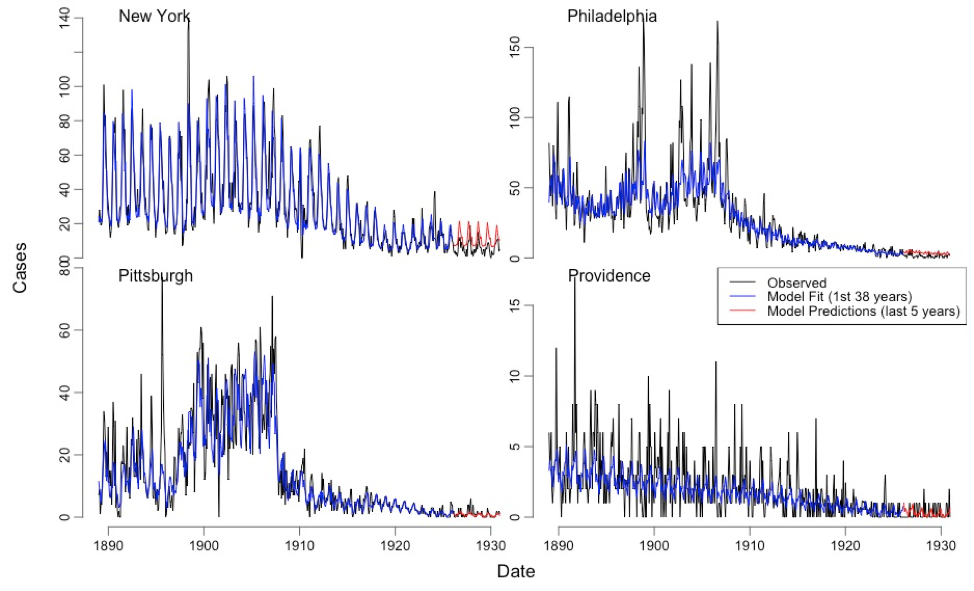

Supplement: S19 Fig — For each city, the TSIR model is fit using the first 38 years of data, then used to predict the last 5 years of data. In each plot, the observed data is shown in black, the model fit to the first 38 years is shown in blue, and the predicted last 5 years is shown in red. (TIF) [file pntd.0008048.s021.tif]

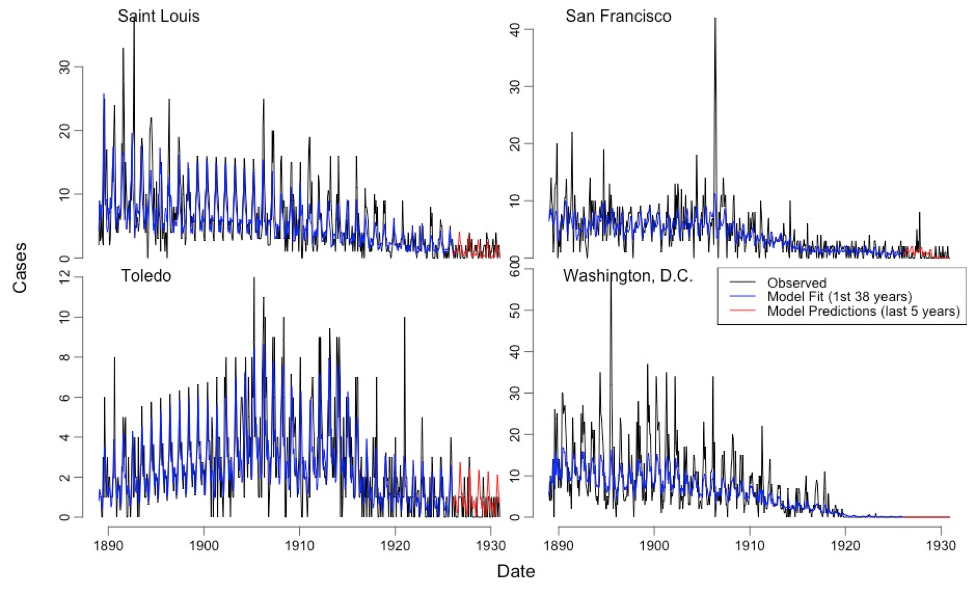

Supplement: S20 Fig — For each city, the TSIR model is fit using the first 38 years of data, then used to predict the last 5 years of data. In each plot, the observed data is shown in black, the model fit to the first 38 years is shown in blue, and the predicted last 5 years is shown in red. (TIF) [file pntd.0008048.s022.tif]

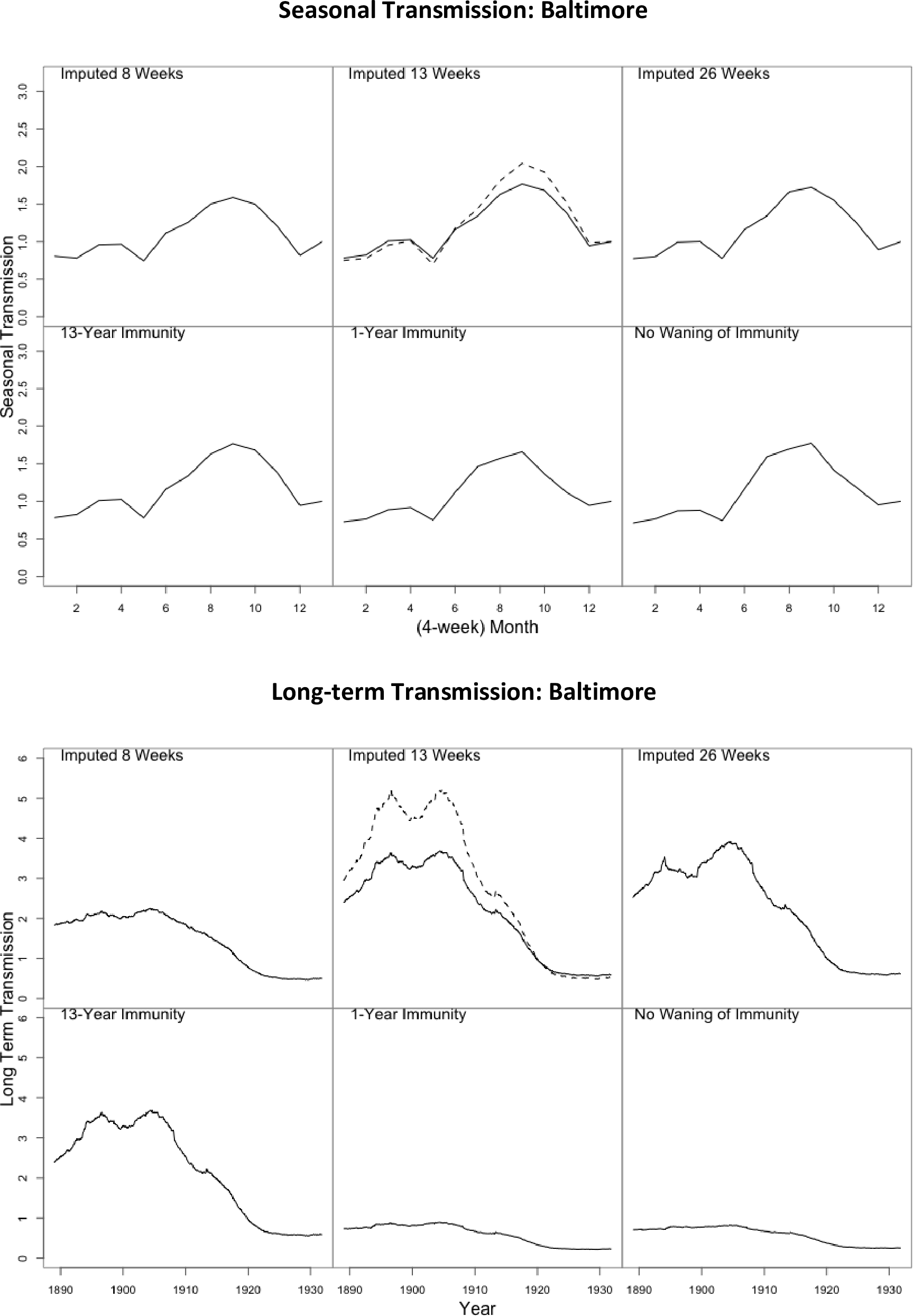

Supplement: S21 Fig — The plot of seasonal and long-term transmission is shown for each city separately. Plots are shown for imputation of missing data (8-, 13-, and 26-week algorithm); addition to all weekly death counts (+1 shown in the solid line in every panel; +0.5 shown in the dashed line in the second panel); and duration of immunity (13-year, 1-year, and no waning of immunity) in the Baltimore data. (TIF) [file pntd.0008048.s023.tif]

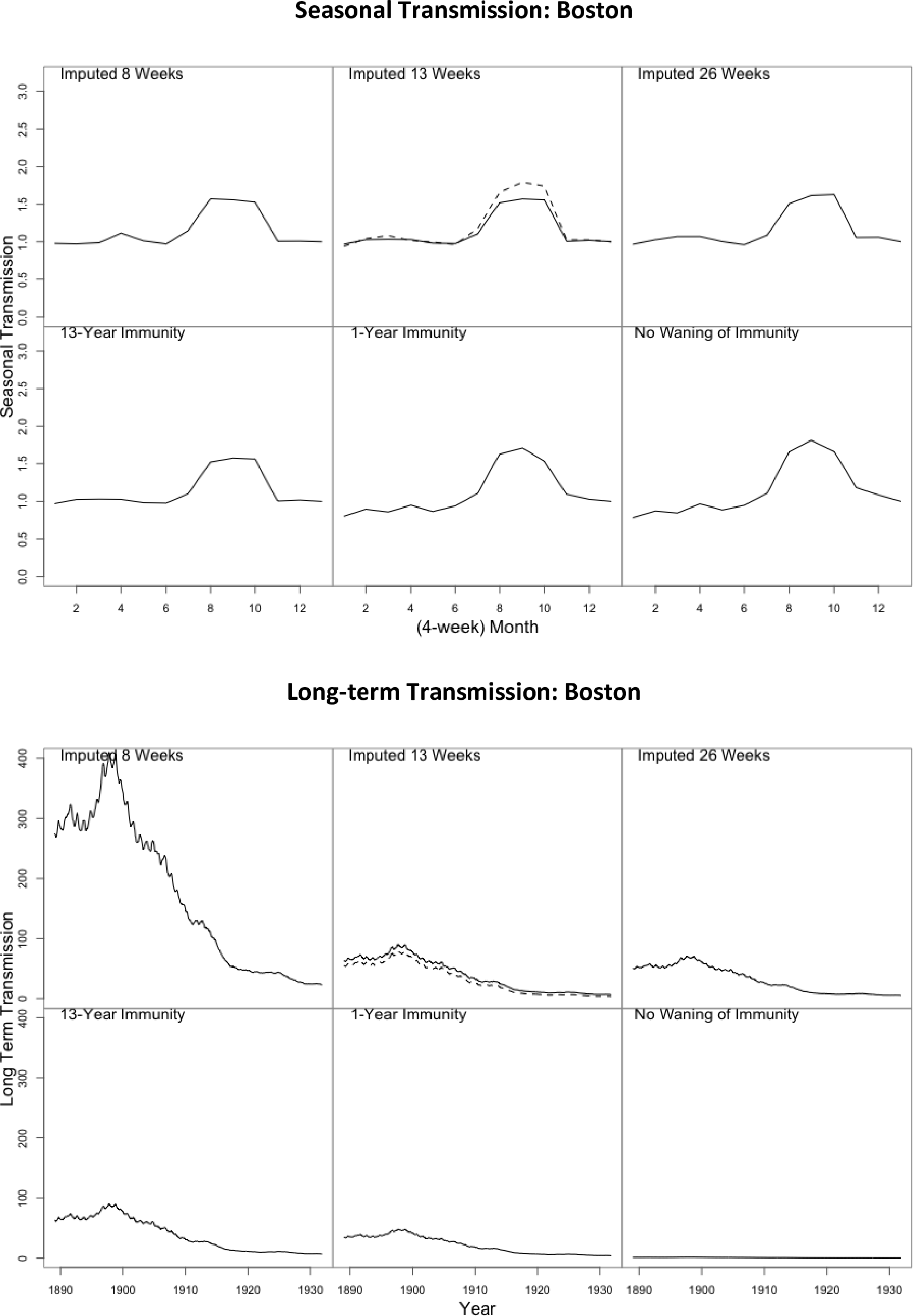

Supplement: S22 Fig — The plot of seasonal and long-term transmission is shown for each city separately. Plots are shown for imputation of missing data (8-, 13-, and 26-week algorithm); addition to all weekly death counts (+1 shown in the solid line in every panel; +0.5 shown in the dashed line in the second panel); and duration of immunity (13-year, 1-year, and no waning of immunity) in the Boston data. (TIF) [file pntd.0008048.s024.tif]

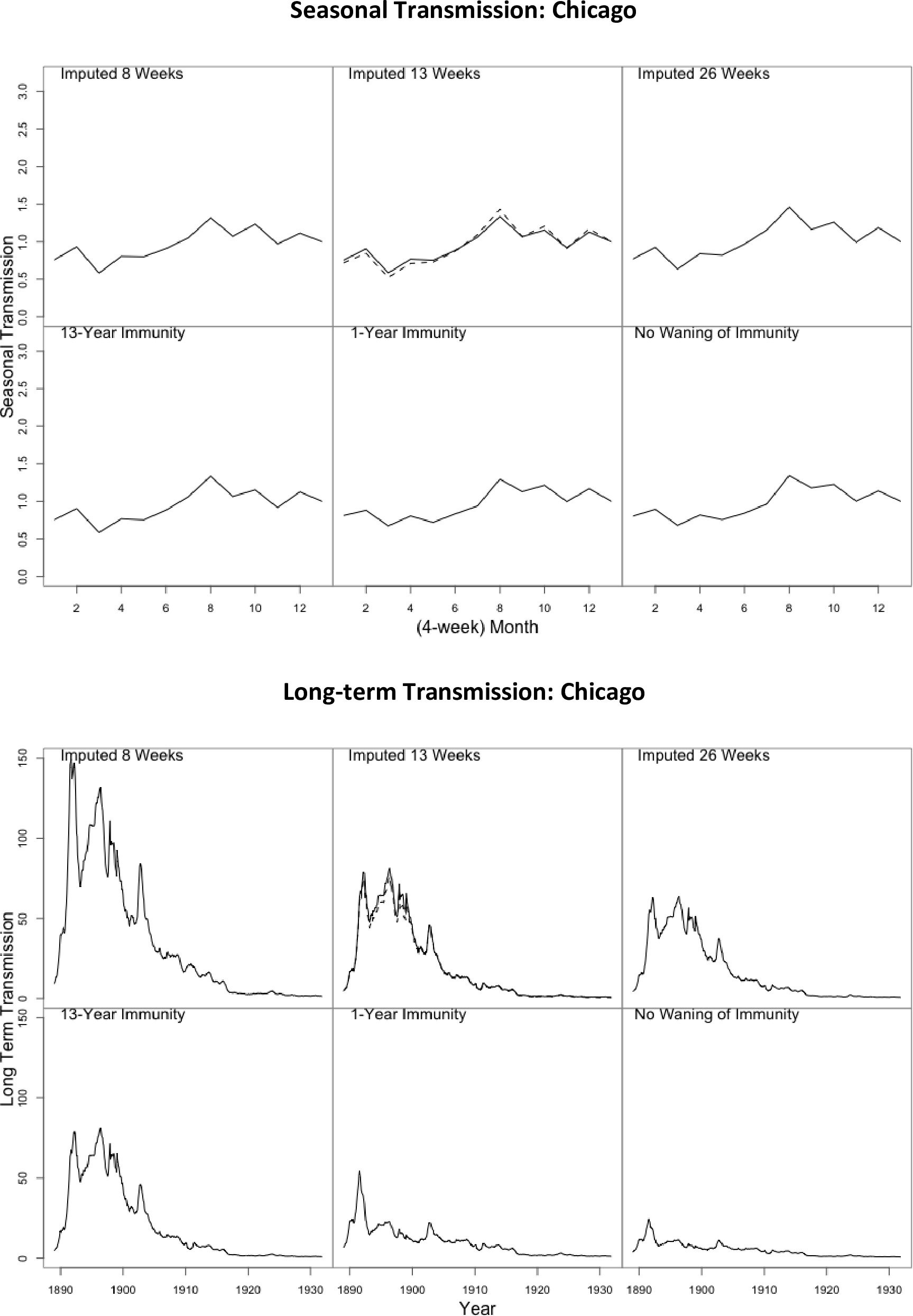

Supplement: S23 Fig — The plot of seasonal and long-term transmission is shown for each city separately. Plots are shown for imputation of missing data (8-, 13-, and 26-week algorithm); addition to all weekly death counts (+1 shown in the solid line in every panel; +0.5 shown in the dashed line in the second panel); and duration of immunity (13-year, 1-year, and no waning of immunity) in the Chicago data. (TIF) [file pntd.0008048.s025.tif]

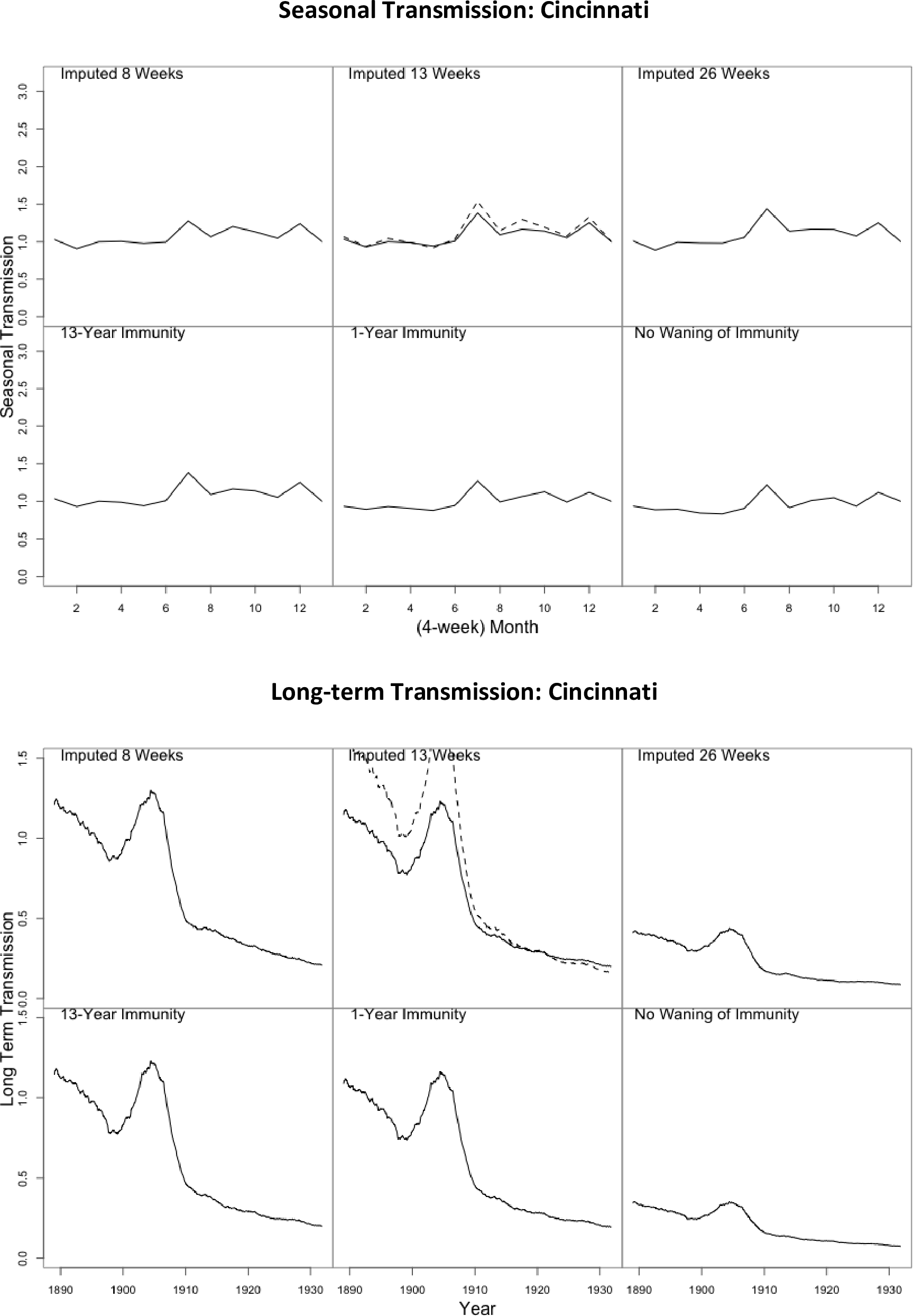

Supplement: S24 Fig — The plot of seasonal and long-term transmission is shown for each city separately. Plots are shown for imputation of missing data (8-, 13-, and 26-week algorithm); addition to all weekly death counts (+1 shown in the solid line in every panel; +0.5 shown in the dashed line in the second panel); and duration of immunity (13-year, 1-year, and no waning of immunity) in the Cincinnati data. (TIF) [file pntd.0008048.s026.tif]

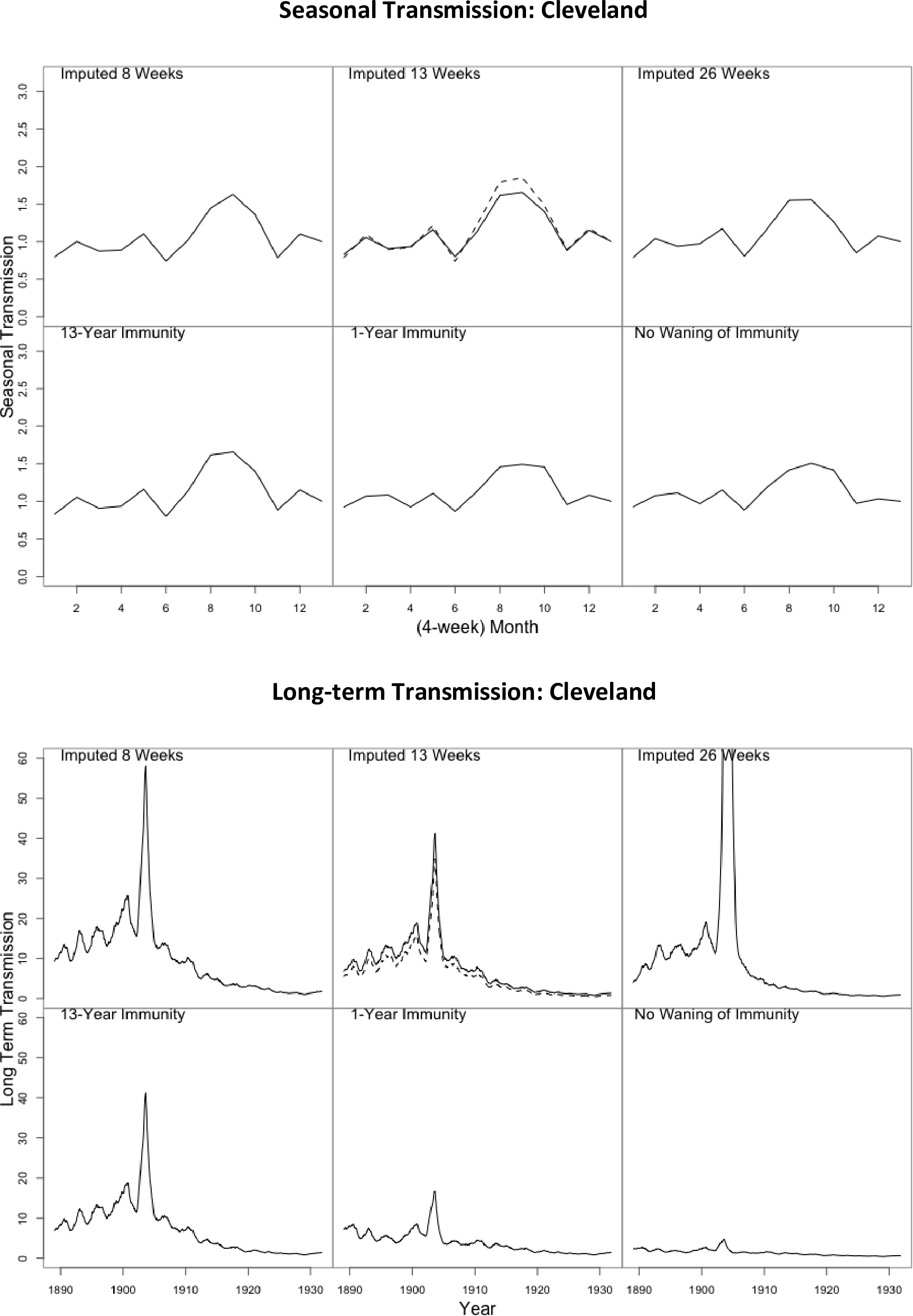

Supplement: S25 Fig — The plot of seasonal and long-term transmission is shown for each city separately. Plots are shown for imputation of missing data (8-, 13-, and 26-week algorithm); addition to all weekly death counts (+1 shown in the solid line in every panel; +0.5 shown in the dashed line in the second panel); and duration of immunity (13-year, 1-year, and no waning of immunity) in the Cleveland data. Note that the 26-week imputation plot is not shown entirely in the plot due to its outlier. (TIF) [file pntd.0008048.s027.tif]

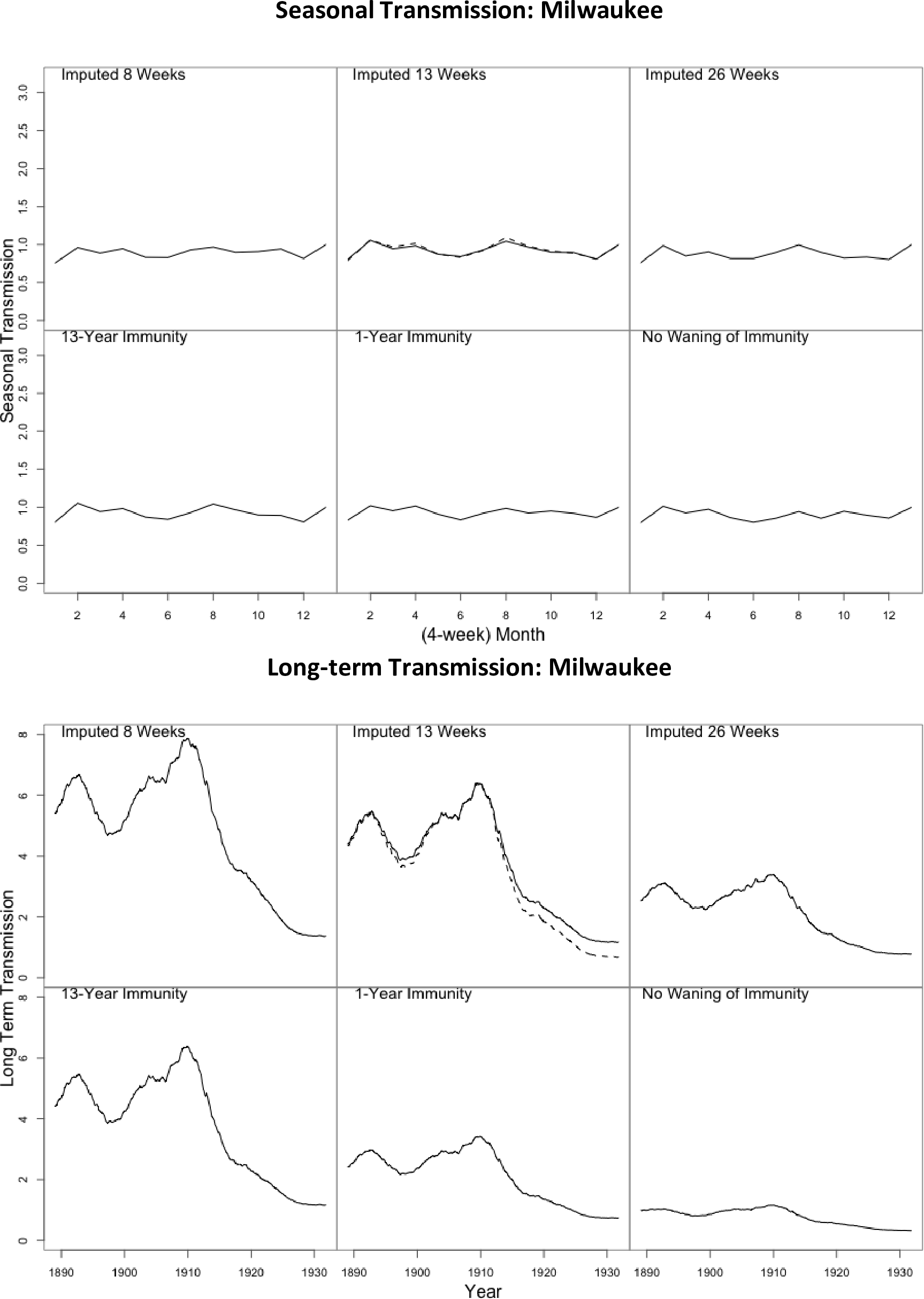

Supplement: S26 Fig — The plot of seasonal and long-term transmission is shown for each city separately. Plots are shown for imputation of missing data (8-, 13-, and 26-week algorithm); addition to all weekly death counts (+1 shown in the solid line in every panel; +0.5 shown in the dashed line in the second panel); and duration of immunity (13-year, 1-year, and no waning of immunity) in the Milwaukee data. (TIF) [file pntd.0008048.s028.tif]

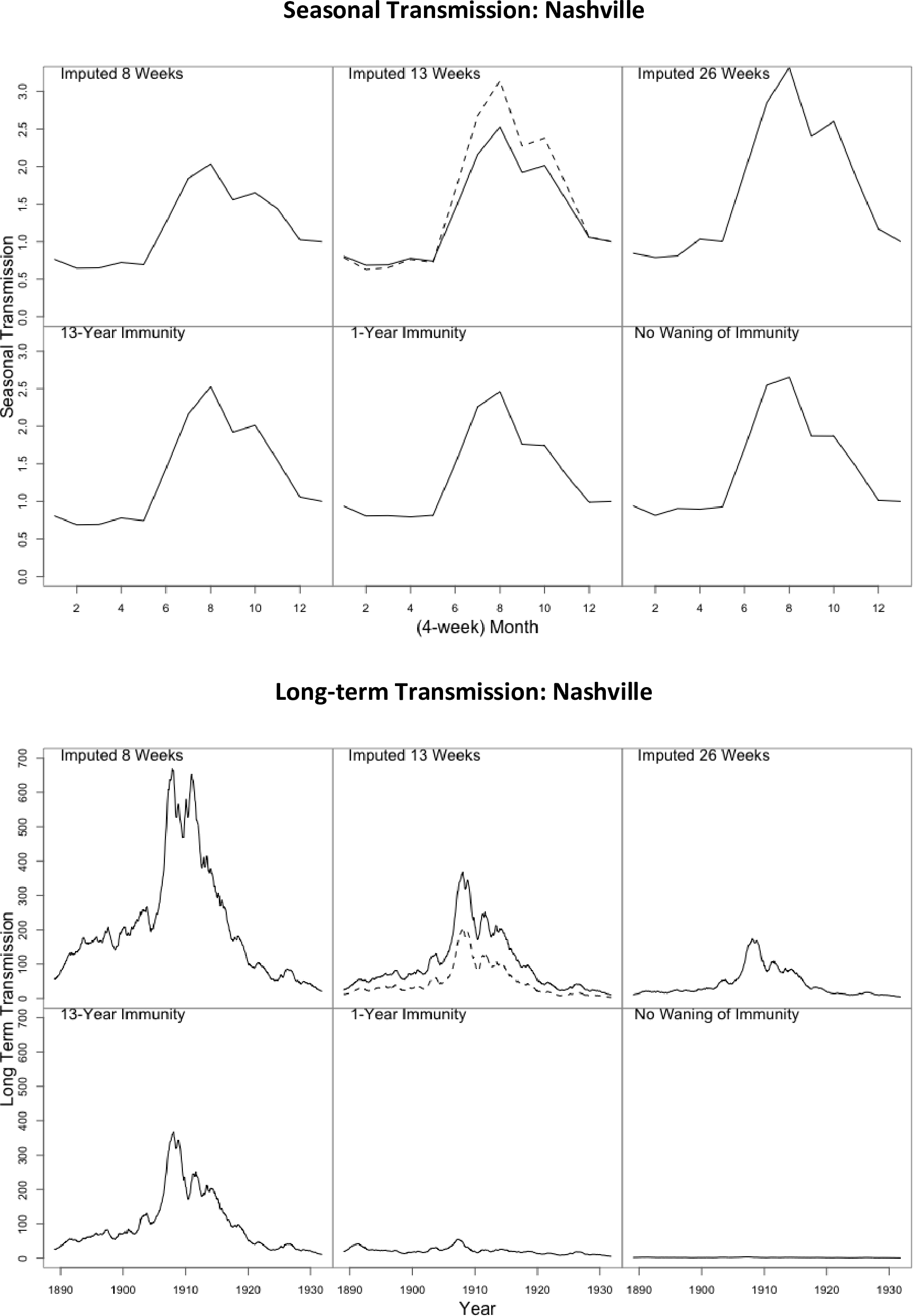

Supplement: S27 Fig — The plot of seasonal and long-term transmission is shown for each city separately. Plots are shown for imputation of missing data (8-, 13-, and 26-week algorithm); addition to all weekly death counts (+1 shown in the solid line in every panel; +0.5 shown in the dashed line in the second panel); and duration of immunity (13-year, 1-year, and no waning of immunity) in the Nashville data. (TIF) [file pntd.0008048.s029.tif]

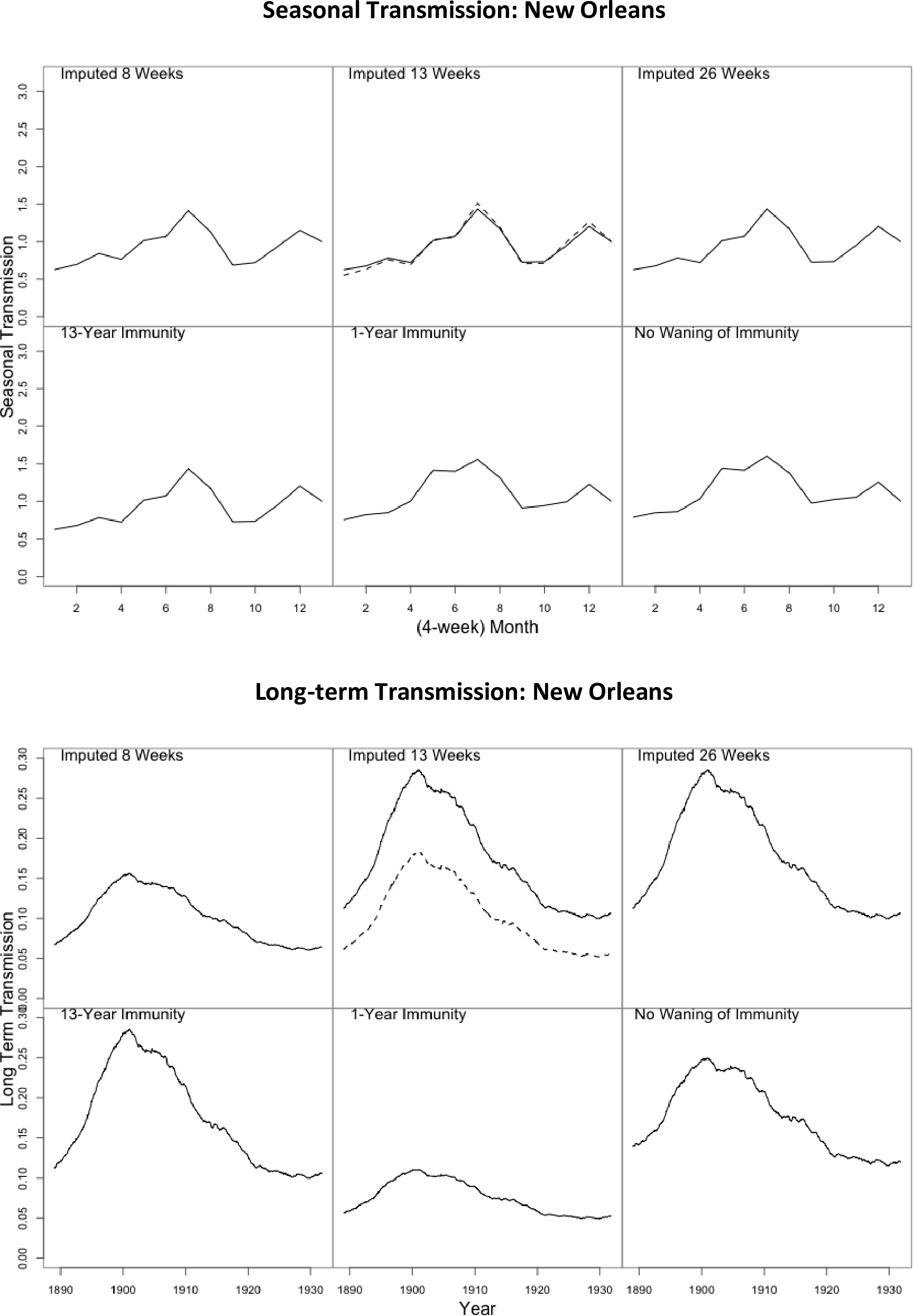

Supplement: S28 Fig — The plot of seasonal and long-term transmission is shown for each city separately. Plots are shown for imputation of missing data (8-, 13-, and 26-week algorithm); addition to all weekly death counts (+1 shown in the solid line in every panel; +0.5 shown in the dashed line in the second panel); and duration of immunity (13-year, 1-year, and no waning of immunity) in the New Orleans data. (TIF) [file pntd.0008048.s030.tif]

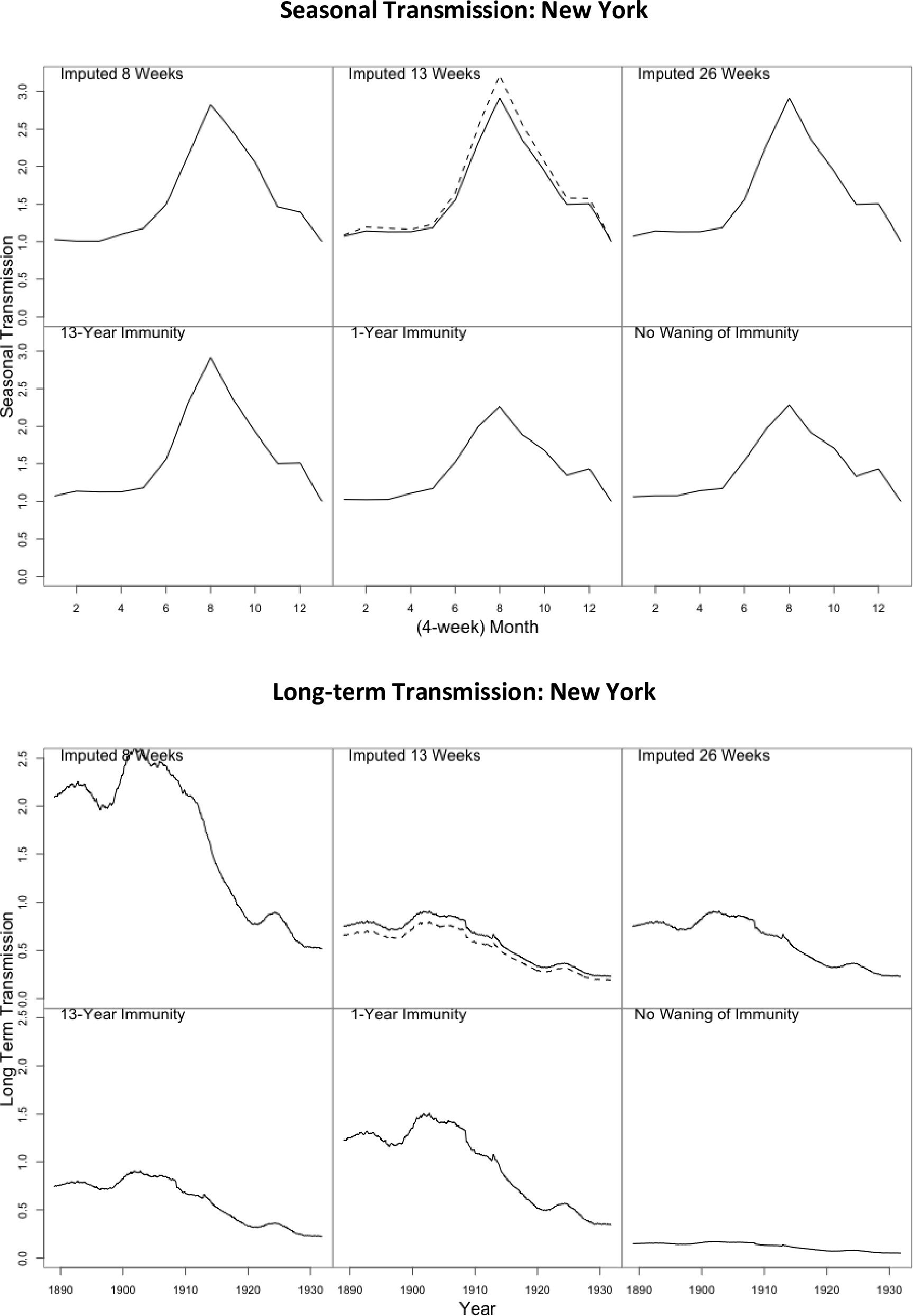

Supplement: S29 Fig — The plot of seasonal and long-term transmission is shown for each city separately. Plots are shown for imputation of missing data (8-, 13-, and 26-week algorithm); addition to all weekly death counts (+1 shown in the solid line in every panel; +0.5 shown in the dashed line in the second panel); and duration of immunity (13-year, 1-year, and no waning of immunity) in the New York data. (TIF) [file pntd.0008048.s031.tif]

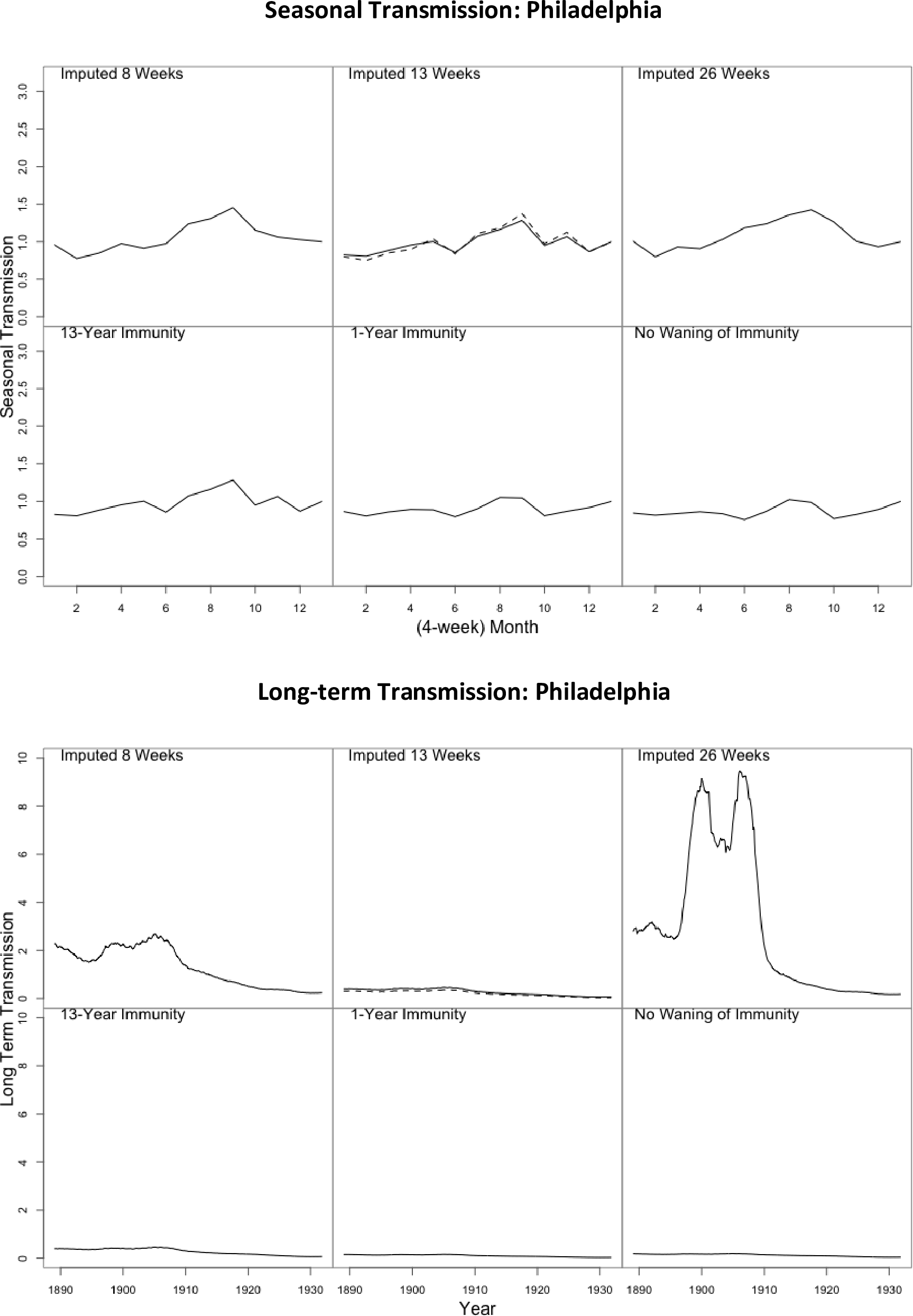

Supplement: S30 Fig — The plot of seasonal and long-term transmission is shown for each city separately. Plots are shown for imputation of missing data (8-, 13-, and 26-week algorithm); addition to all weekly death counts (+1 shown in the solid line in every panel; +0.5 shown in the dashed line in the second panel); and duration of immunity (13-year, 1-year, and no waning of immunity) in the Philadelphia data. (TIF) [file pntd.0008048.s032.tif]

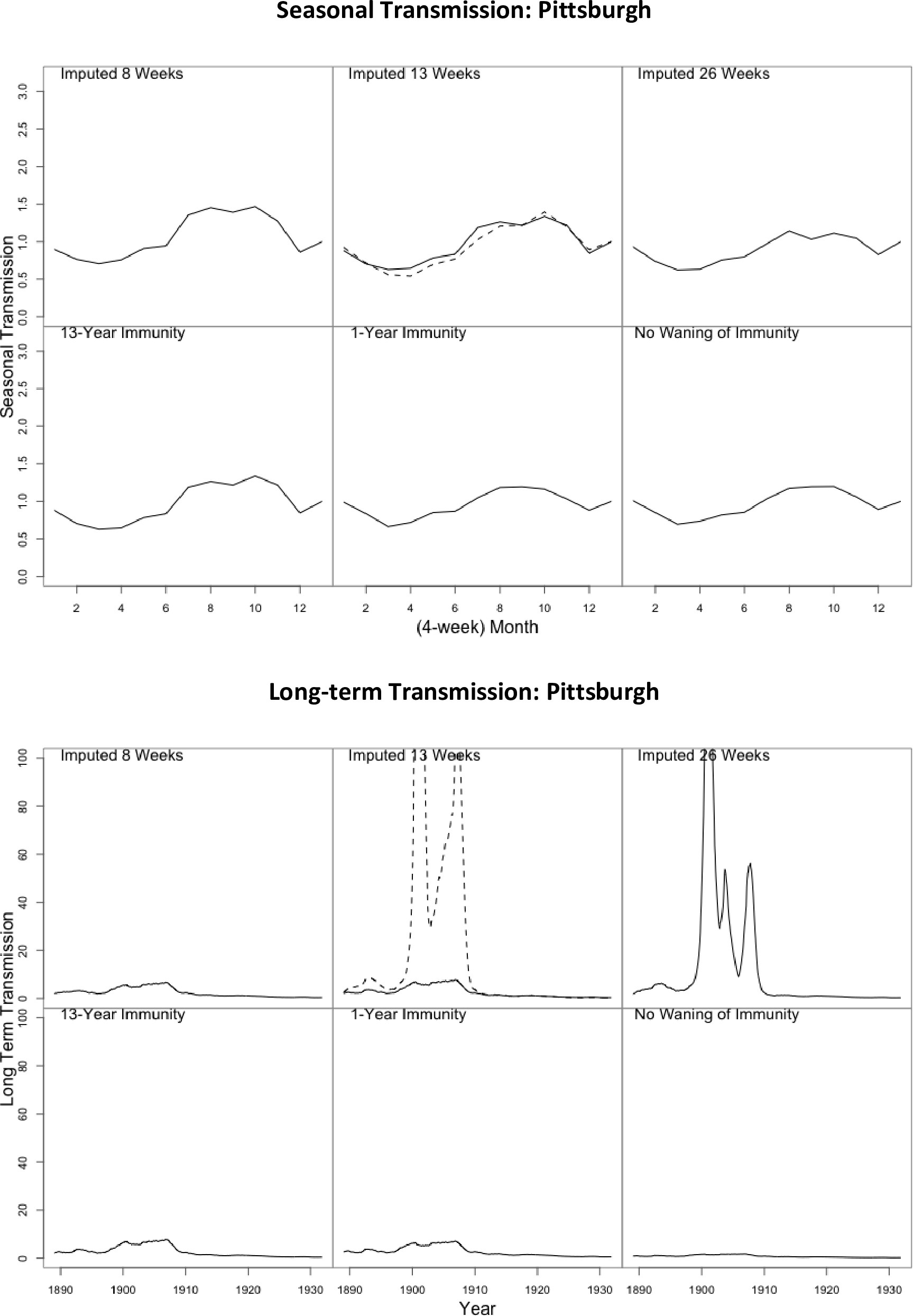

Supplement: S31 Fig — The plot of seasonal and long-term transmission is shown for each city separately. Plots are shown for imputation of missing data (8-, 13-, and 26-week algorithm); addition to all weekly death counts (+1 shown in the solid line in every panel; +0.5 shown in the dashed line in the second panel); and duration of immunity (13-year, 1-year, and no waning of immunity) in the Pittsburgh data. Note that the imputed 13-week algorithm (+0.5) and the imputed 26-week algorithm (+1) are not shown entirely in the plots due to outliers. (TIF) [file pntd.0008048.s033.tif]

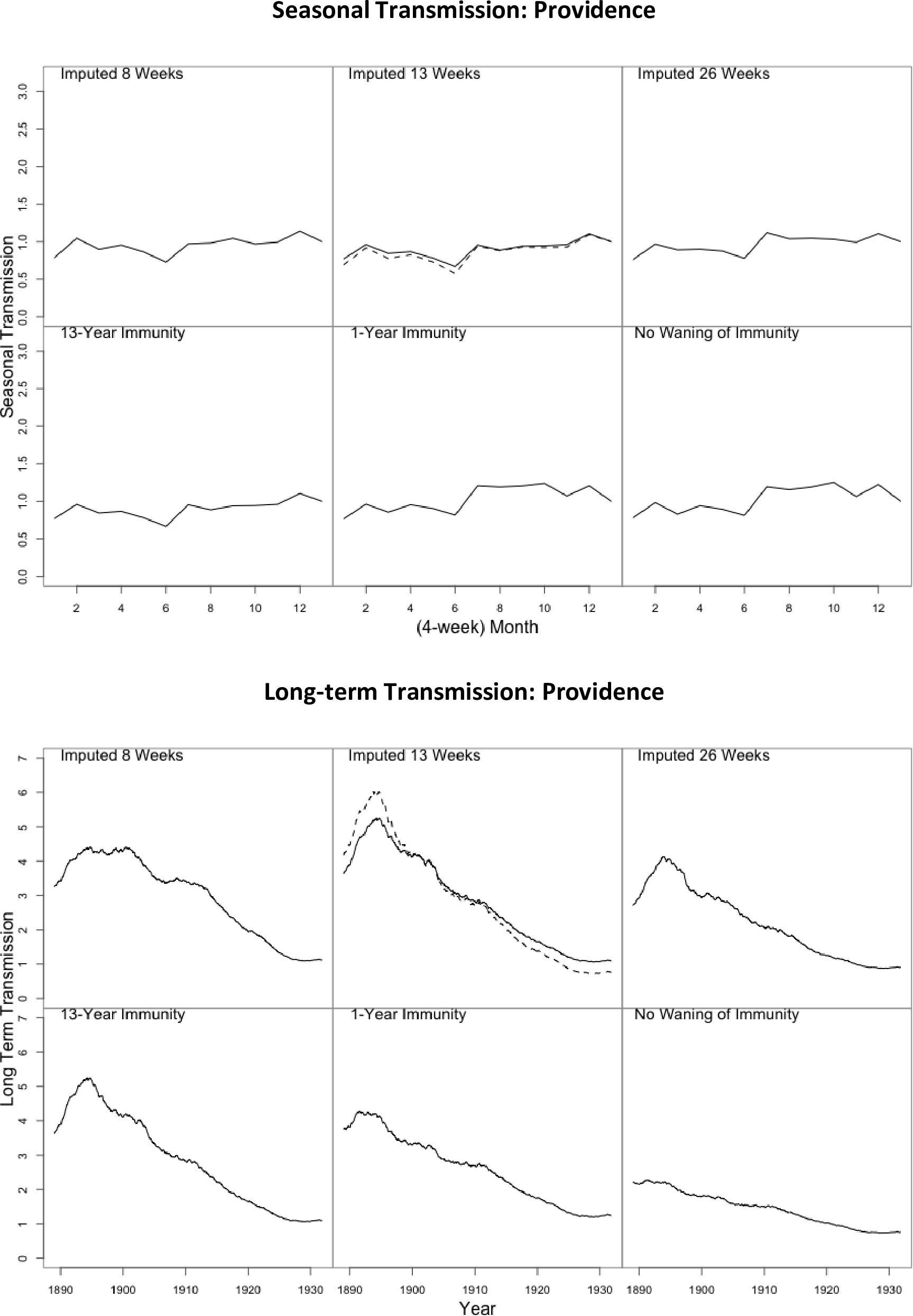

Supplement: S32 Fig — The plot of seasonal and long-term transmission is shown for each city separately. Plots are shown for imputation of missing data (8-, 13-, and 26-week algorithm); addition to all weekly death counts (+1 shown in the solid line in every panel; +0.5 shown in the dashed line in the second panel); and duration of immunity (13-year, 1-year, and no waning of immunity) in the Providence data. (TIF) [file pntd.0008048.s034.tif]

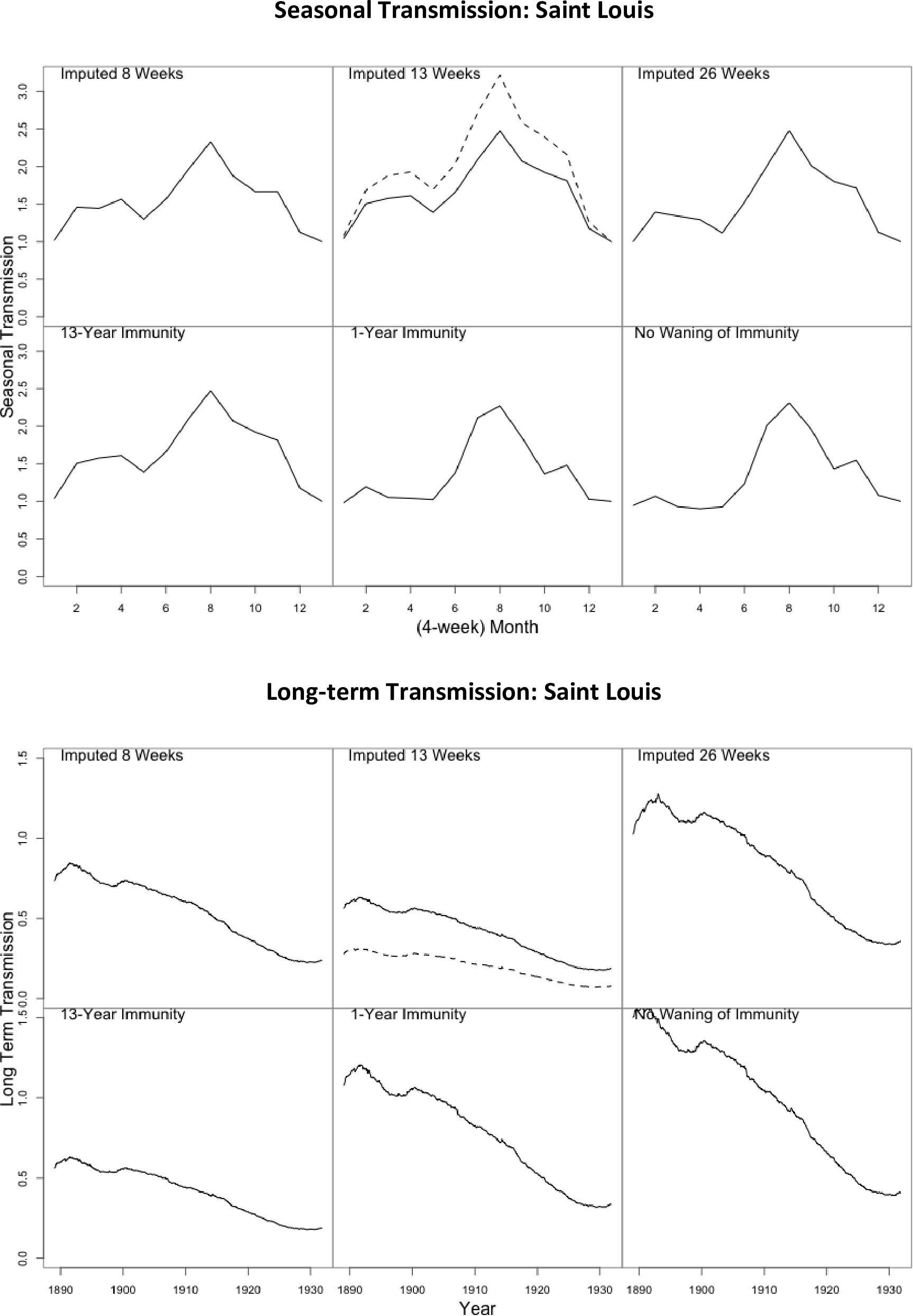

Supplement: S33 Fig — The plot of seasonal and long-term transmission is shown for each city separately. Plots are shown for imputation of missing data (8-, 13-, and 26-week algorithm); addition to all weekly death counts (+1 shown in the solid line in every panel; +0.5 shown in the dashed line in the second panel); and duration of immunity (13-year, 1-year, and no waning of immunity) in the Saint Louis data. (TIF) [file pntd.0008048.s035.tif]

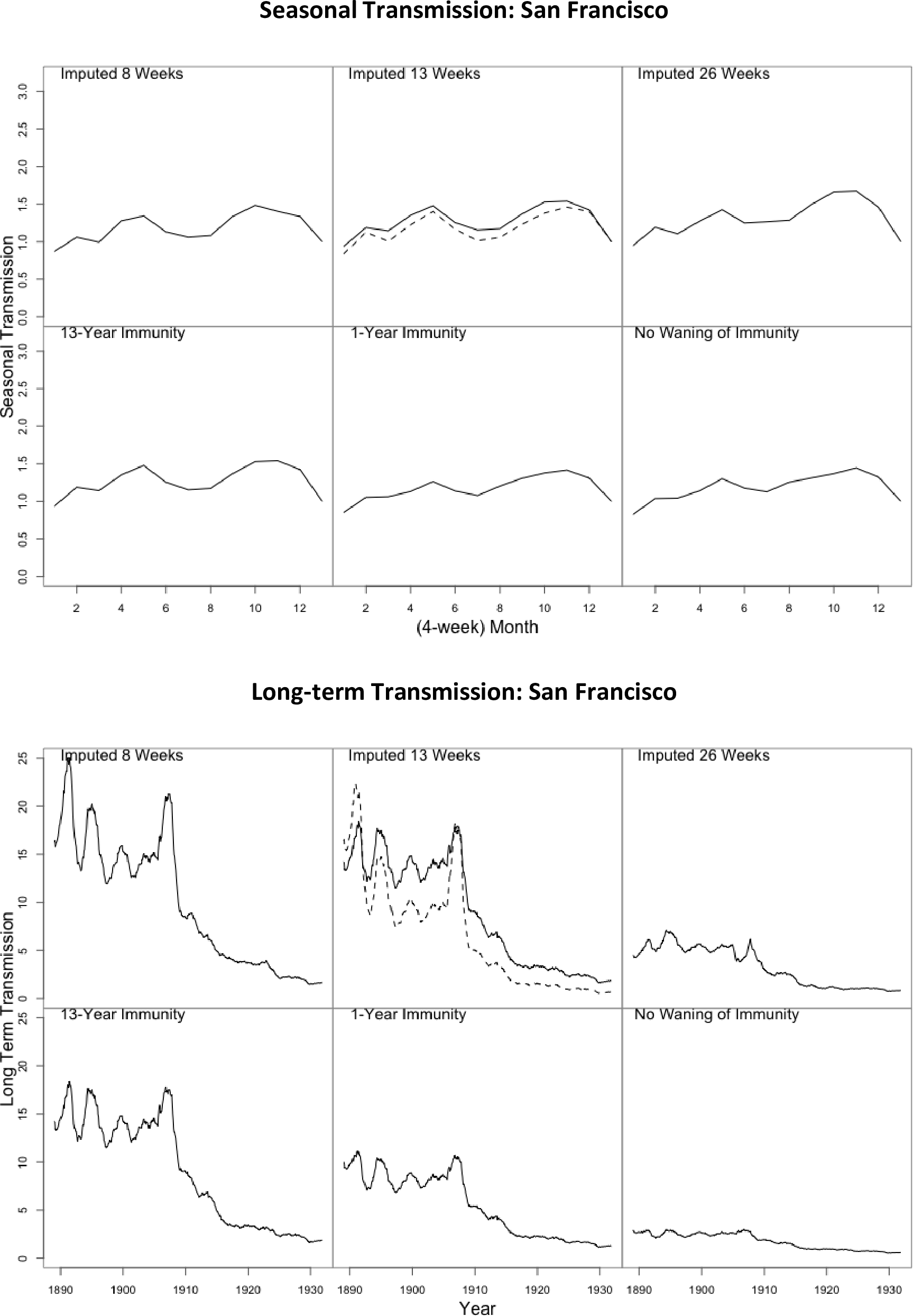

Supplement: S34 Fig — The plot of seasonal and long-term transmission is shown for each city separately. Plots are shown for imputation of missing data (8-, 13-, and 26-week algorithm); addition to all weekly death counts (+1 shown in the solid line in every panel; +0.5 shown in the dashed line in the second panel); and duration of immunity (13-year, 1-year, and no waning of immunity) in the San Francisco data. (TIF) [file pntd.0008048.s036.tif]

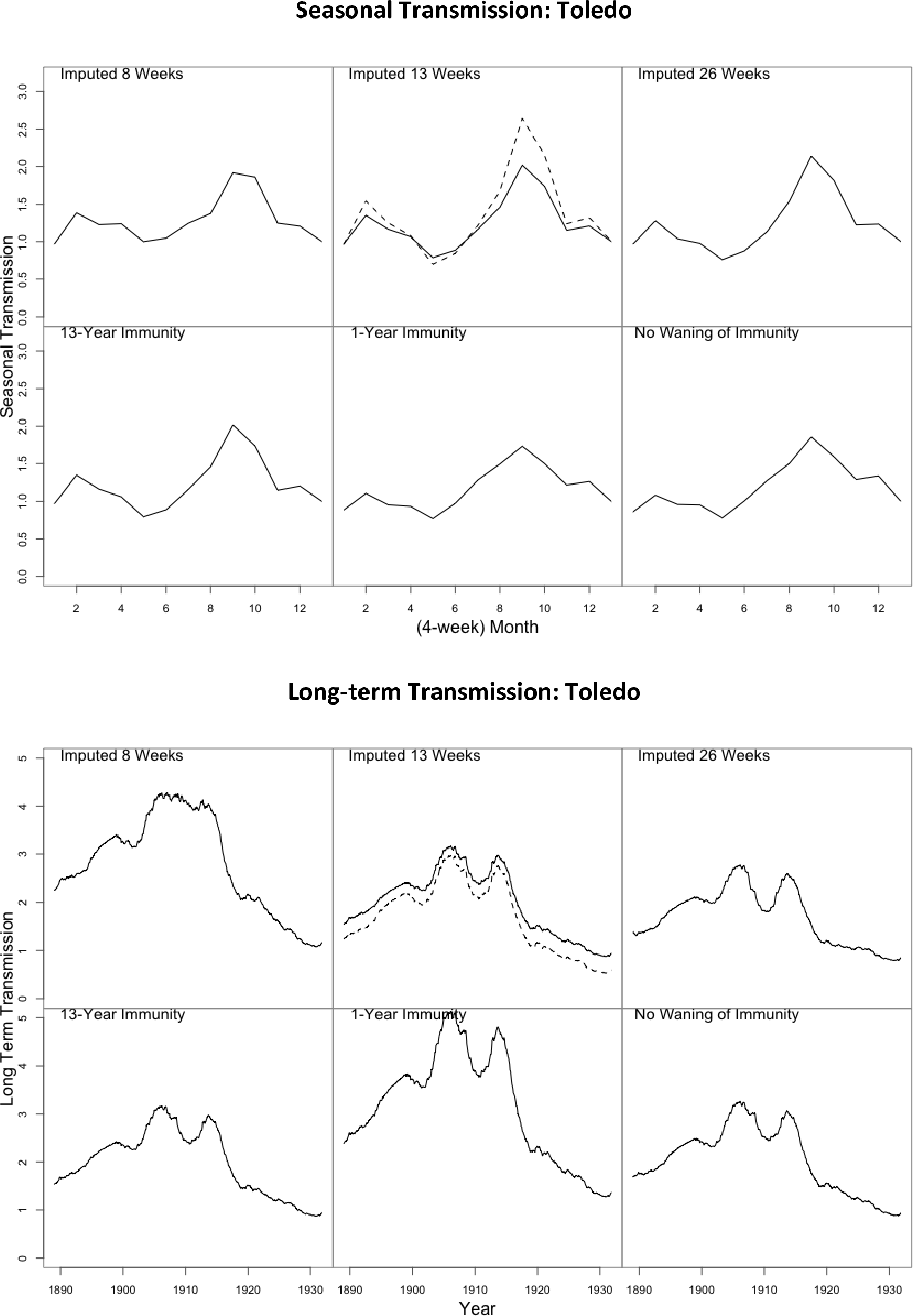

Supplement: S35 Fig — The plot of seasonal and long-term transmission is shown for each city separately. Plots are shown for imputation of missing data (8-, 13-, and 26-week algorithm); addition to all weekly death counts (+1 shown in the solid line in every panel; +0.5 shown in the dashed line in the second panel); and duration of immunity (13-year, 1-year, and no waning of immunity) in the Toledo data. (TIF) [file pntd.0008048.s037.tif]

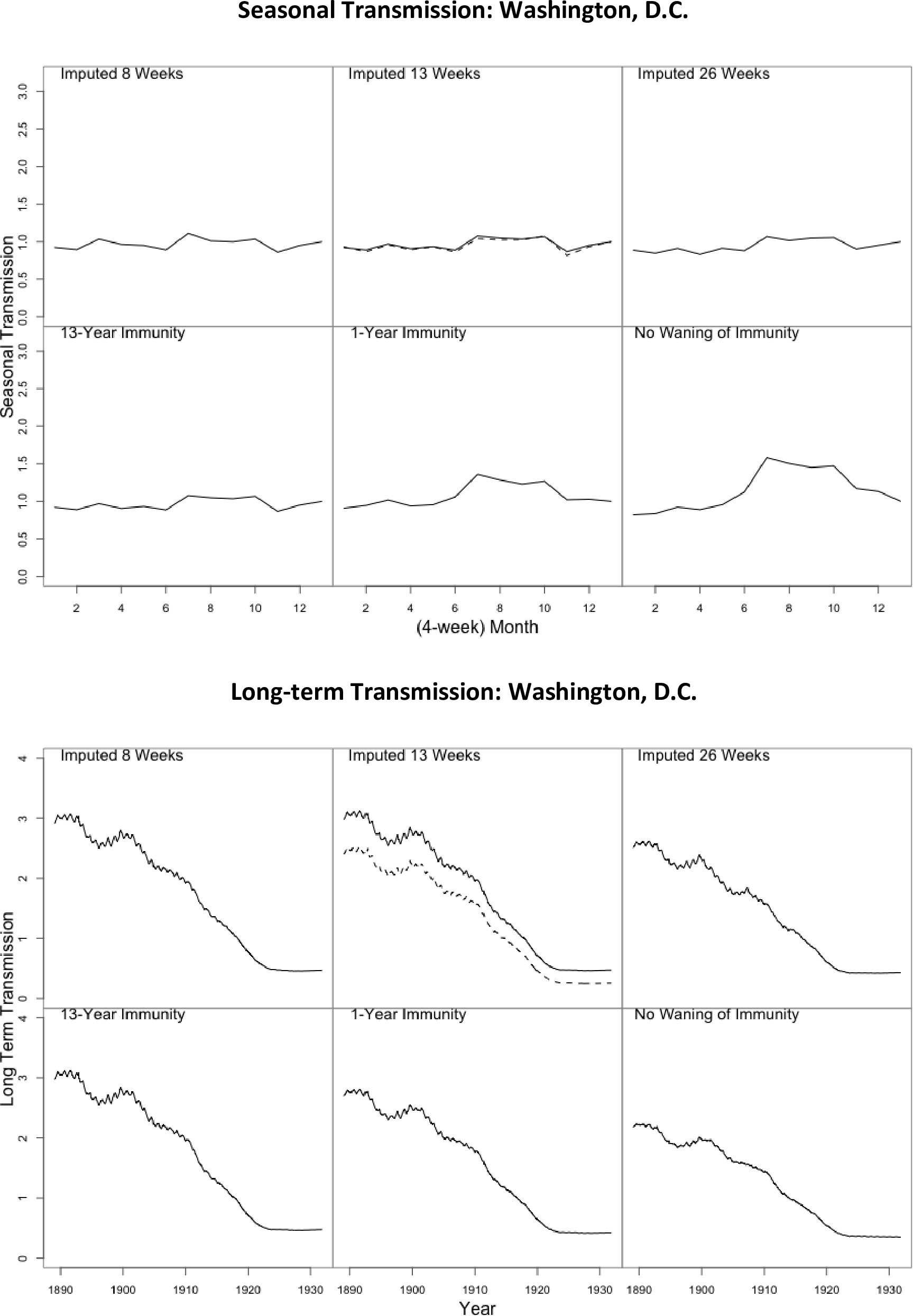

Supplement: S36 Fig — The plot of seasonal and long-term transmission is shown for each city separately. Plots are shown for imputation of missing data (8-, 13-, and 26-week algorithm); addition to all weekly death counts (+1 shown in the solid line in every panel; +0.5 shown in the dashed line in the second panel); and duration of immunity (13-year, 1-year, and no waning of immunity) in the Washington, D.C. data. (TIF) [file pntd.0008048.s038.tif]
