# Supplementary material for: Changes in historical typhoid transmission across 16 U.S. cities, 1889-1931: Quantifying the impact of investments in water and sewer infrastructures
Source: PLoS Negl Trop Dis. 2020 Mar 18;14(3):e0008048. doi: 10.1371/journal.pntd.0008048 (PMC7105137; doi:10.1371/journal.pntd.0008048)
Supplement: Attachment — Submitted filename: review.docx [file pntd.0008048.s049.docx]

This study provides a historical perspective on typhoid transmission 1889 to 1931 across 16 U.S. cities. The goals of the paper were to characterize typhoid seasonality and examine the impact of citywide investments in water and sanitation infrastructure on long-term typhoid transmission and seasonality. While I cannot comment on the novelty of these findings for typhoid epidemiology, I have suggestions and concerns related to the methods and the level of evidence provided by the results; the authors need to clarify many points before I can fully evaluate the reasonableness of the conclusions. Once these points are further described, the results of this study could have the potential to elucidate mechanisms behind typhoid’s historical decline in the U.S. and provide additional epidemiological evidence for the link between improved water and sanitation and reduced typhoid transmission and burden.

Major comments:

Data Inputs: There needs to be more detail and justifications for various data processing steps; at the very least, this information should be put in a supplement file. In particular:

- Lines 115-116 says that cubic splines were used to extrapolate weekly population estimates. Is this only for the consolidated New York City population data, or for all cities?
- Were the financial data adjusted for inflation, and if yes, by what method? If they were not adjusted for inflation, there needs to be some justification for why we should believe that the per capita investment increases over time are not spurious. Given that many of the results (e.g., Figure 2 and Table 3) hinge on demonstrating the relationship between increasing investments and declining typhoid transmission, this seems to be a critical point in need of clarification.
- Can the authors provide more information about how the “first intervention” was defined? Would small investments count towards this measure? Or would it need to be an intervention that affected the entire city? I’m not sure that the interventions are aligned with large increases to water supply outlays in Figure S7. It makes me wonder if the financial data may not appropriately capture these investments because a large investment would be paid off with loans over time.
- Given the goal of characterizing typhoid seasonality, there should be greater discussion of the methods used to impute typhoid mortality data. There are several imputation methods available in *imputeTS* and there should be a brief text description of the general approach. In particular, I would want to know what, if any, seasonality assumptions were used in these imputation procedures, as this could affect the reliability of the seasonality results. In addition, the authors should clarify which non-missing data were used to impute the missing values (e.g., The remaining available time series? Only surrounding years?).
- In a similar vein, what method was used to approximate the generation interval (Line 140)? More information should be provided.

Statistical Methods: I found the sections describing the TSIR models somewhat difficult to follow. Some of the terms were not well defined and I don’t believe the description explicitly provides enough information to understand how the equations come together. I might suggest a broader restructuring of this section where the main model equation is presented first; complex components in that main equation can be represented as generic functions, which are then subsequently explained with more detail. If the authors explicitly stated which parameters they aimed to estimate with the model at the beginning of the section, the readability of this section may also be improved. For instance, I think lines 189-191 describe the reason for fitting the TSIR model and seem a bit out of place in the next section.

- I’m confused about why only the first ten years of data were used for the TSIR model described starting in line 158 when much of the rest of the text reports model results for the entire time series for the first 38-year period. Is this a typo or was there some separate initialization model?
- Line 163: S^bar is the mean susceptible population – mean of what? Deviation (D_0) of what?
- While there is a bit more detail about the transmission parameters in S1 text, I think it would still be helpful to have some nested time-indexing (e.g., something like beta_{seas, m[k]}, where m is the month of time k) in the model equations for the beta parameters. It seems that there is essentially one beta_seas per fixed month, but I’m not sure about how many beta_lt parameters there are for each model. Figure 2 implies that there might be one parameter per time point?
- Throughout this section, I was wondering why waning immunity did not feature into any of the equations for I_t and then I was not quite sated by the description of immunity in the last paragraph of this section. What quantitative assumptions are you using for immunity and where are the primary references? As I mentioned above, it might be less confusing for readers if this section starts with a description of the full model and breaks down the components into finer detail, as opposed to building it up from the simplest form of a TSIR.
- Did the authors perform some sort of statistical test to determine that the pre- and post-intervention transmission parameters were different? Or was this through visual inspection of Figure S3?

Results

- It is hard for me to see the results from lines 231-235 visualized in Figure 1. Perhaps the peak transmission months for each city could be highlighted in some way and the x-axis could provide more detailed labels? I guess the city-level panels represent the median estimate for beta_seas?
- The immediate question that follows Figure 1 is whether the cities with similar seasonality have similar precipitation/temperature patterns (as highlighted by previous works), or whether they are geographically clustered. While there is some treatment of these hypotheses in the discussion, is there any possibility of examining this directly with real data?
- Was there any formal statistical comparison between cities with different water source types? The authors state that there are differences, but the variability in estimates are shown neither at the city level nor for the summarized panel by water source type in Figure 1 so it is hard to assess this claim.
- Also, the peak amplitude for the median curve is shifted later than the peak amplitude for all three water source type curves (which appears aligned). Does that make sense?
- Can the authors also report the alpha estimates for each city in Table S3? This can serve as another gut check that the TSIR models make sense (as well as have reasonable fits).
- The Results section is heavily focused on reporting the univariate financial data regression results, when for me this feels like the least compelling part of the analysis. I’m not sure how informative or generalizable it is to say that one variable was correlated in two cities, another one was correlated in a different two cities, etc. The results might be more robust and generalizable to current typhoid control efforts if the authors could say something more broadly about the impacts of investments in water versus sewer infrastructure, or investments in large overhauls versus regular maintenance. Was there some reason that the data across cities could not be combined in a hierarchical model for each financial variable? At the very least, there should be some significance-level correction for multiple testing across so many variables and cities.
- I think it will make sense to describe the model validation procedure somewhere in the Methods section (maybe a separate sub-section?) since these results are reported in the main text.

I don’t feel quite convinced about the conclusion that the results provide evidence that water and sewer systems investments contributed to typhoid’s decline. I understand the general limitations of a historical study such as this, but the authors felt that the pre- and post-intervention TSIR models were sufficiently similar to present only model results for the entire time period. For instance, was there a formal analysis to compare beta_lt and beta_seas similarities before and after the first intervention? (I believe that only the seasonal transmission parameter results were discussed in this section, and it’s not clear to me how they were compared.) If the single biggest potential changepoint (time of first intervention) did not produce differences in both sets of transmission parameters, why should we then defer to the more simplistic univariate regression financial results when drawing the conclusions? There are many other untested hypotheses that might explain the associations between individual financial variables and long-term transmission trends.

There were several places where the text explains that sensitivity analyses were performed on immunity duration assumptions and consecutive missing data for imputation, but these results do not appear to be anywhere in the supplement (e.g., last paragraph of the Study Design, Data, and Variables section, last paragraph of the Results, S1 text). I think readers will want to see the specifics of the sensitivity analyses and their results.

Minor comments:

- Lines 111-115: The language describing the New York City population processing could be cleaned up.
- I think the categories listed in lines 122-123 should match the labels in Table 1. Also, I was confused about the “eight” variables described in line 122. Which variables are these?
- Line 163: Define k
- Line 164: I think the summation term for I_k should go from k = 0 to t-1
- Line 166: It might be better to remove the equation in parentheses here and write out the description in text (e.g., “…underreporting factor rho, where the true infections at time t (I_t) is calculated as observed deaths (Y_t) divided by rho…”). Y_t should also be defined explicitly here.
- Line 192: I think this must be the R Studio version number and not the R version number.
- Line 195-196: I do not understand this sentence: “Complete case analysis was assumed for missing financial variable data”
- What are the units for the secondary y-axis in Figure 2?
