# Supplementary material for: Changes in historical typhoid transmission across 16 U.S. cities, 1889-1931: Quantifying the impact of investments in water and sewer infrastructures
Source: PLoS Negl Trop Dis. 2020 Mar 18;14(3):e0008048. doi: 10.1371/journal.pntd.0008048 (PMC7105137; doi:10.1371/journal.pntd.0008048)
Supplement: Attachment — Submitted filename: review.docx [file pntd.0008048.s050.docx]

The revision of this manuscript on the impact of water and sewer infrastructures on historical typhoid transmission is much improved, particularly in relation to the clarity of the methods and transparency in the results. I still have some concerns about the methods and validity of the TSIR model that need to be addressed before the paper can be published.

Major comments:

- The Methods on the hierarchical regression models for long-term typhoid transmission and financial investments should have more details on how it is hierarchical, either through explicit equations or a description of the model covariates, and how the models were fit.
- It is not appropriate to use AIC to compare models with different model outcomes (e.g., log-transformed versus non-transformed). In this circumstance, I think it would be okay to rely on exploratory analyses to justify the functional relationship between the response and covariates and skip model selection.
- The newly reported heterogeneous mixing coefficient estimates for the TSIR model are very low (S3 Table), including some values that are negative. This seems problematic and I’m not sure under what justification the authors assert that the approach remains valid. If there is indeed some interaction with the beta transmission terms as the authors suggest, I wonder that they did not try constraining the beta terms in some way as suggested by R1. While fixing beta_seas[13] at 1 reduces the degrees of freedom, perhaps there are identifiability issues between beta_seas and beta_lt that are pushing the model into an unrealistic parameter space.

Minor comments:

- I might recommend using a different notation for births since you already have two beta transmission parameters.
- Line 363: I think this should be “S17-S20 figs.” Maybe some of the other supplemental material numberings are off too?
