# Supplementary material for: Changes in historical typhoid transmission across 16 U.S. cities, 1889-1931: Quantifying the impact of investments in water and sewer infrastructures
Source: PLoS Negl Trop Dis. 2020 Mar 18;14(3):e0008048. doi: 10.1371/journal.pntd.0008048 (PMC7105137; doi:10.1371/journal.pntd.0008048)
Supplement: Attachment — Submitted filename: review response 12.15.19.docx [file pntd.0008048.s051.docx]

12.15.2019

Maile T. Phillips

P.O. Box 208034

60 College St.

New Haven, CT 06520-8034 USA

E-mail: maile.phillips@yale.edu

**PNTD-D-19-00963**

Dear Dr. Azman,

We would again like to thank both you and the reviewers for the helpful suggestions for our manuscript.

In response to the second round of comments, we have now added more details to describe the hierarchical regression model fit, and added subheadings in the Results section. We have responded to each comment individually and noted where our changes are reflected in the text.

Thank you again for considering this revision. We believe our changes adequately address the issues raised by the reviewers and look forward to hearing your response.

Sincerely,

Maile T. Phillips

Detailed Response:

**REVIEWER 1:**

I think the revised manuscript is a lot easier to read, with more streamlined results and a clearer message. The new supporting Tables and Figures are helpful.

In the last paragraph of the Introduction (lines 96-101), the mathematical modeling is mentioned but not the statistical analyses. It would be good to mention both approaches since they address different parts of your objectives.

RESPONSE:

We have changed the first sentence in that paragraph to read, “In this study, we developed mathematical and statistical models to examine seasonal and long-term trends…” (lines 96-97).

I can't see a caption in the Table S4 doc file.

RESPONSE:

Thank you for catching this. We have added the table title and caption to the supplementary file.

Caption for Table S7 refers to "each city".

RESPONSE:

We have removed “each city” from the caption.

For the caption of Figure S5, mention what the dashed black line is.

RESPONSE:

We have added “(dashed black line)” after the susceptible population in the caption.

**REVIEWER 2:**

I could not find any point-by-point response from authors to reviewers' comments.
However, I am quite satisfied with the additional elements and justifications provided in the revised manuscript.

To my opinion, the new version of the manuscript would benefit from a few additional minor modifications.
Additional details should be added in the Methods section to describe the hierarchical regression analyses. It seems that Authors used mixed models with fixed and random effect but do not clearly explain it.

RESPONSE:

We have added in more details to describe the hierarchical model in lines 250-258.

The result section is quite dense and it could thus be easier to read after including subheadings.

RESPONSE:

Thank you for this suggestion. We have added several subheadings to the results section.

The new Results section includes a few sentences that may be displaced in the Discussion (ex. lines 289-290;  lines 299-302).

RESPONSE:

We moved the line about our hypothesis about water source type to the part of the Discussion where we discuss possible explanations for that variation (now lines 413-415). However, we believe the sentence about differences in the seasonality typhoid mortality versus transmission rates pre- vs post-intervention (now lines 321-324) is an important finding of our preliminary analysis that helped to motivate our subsequent approach, and therefore we think it is most appropriate in the Results.

**REVIEWER 3:**

The revision of this manuscript on the impact of water and sewer infrastructures on historical typhoid transmission is much improved, particularly in relation to the clarity of the methods and transparency in the results. I still have some concerns about the methods and validity of the TSIR model that need to be addressed before the paper can be published.

Major comments:

- The Methods on the hierarchical regression models for long-term typhoid transmission and financial investments should have more details on how it is hierarchical, either through explicit equations or a description of the model covariates, and how the models were fit.

RESPONSE:

We have added in more details to describe the hierarchical model in lines 250-258.

- It is not appropriate to use AIC to compare models with different model outcomes (e.g., log-transformed versus non-transformed). In this circumstance, I think it would be okay to rely on exploratory analyses to justify the functional relationship between the response and covariates and skip model selection.

RESPONSE:

We have changed this part of the methods section and now refer to choosing the best model formulation based on exploratory analyses (lines 250-252).

- The newly reported heterogeneous mixing coefficient estimates for the TSIR model are very low (S3 Table), including some values that are negative. This seems problematic and I’m not sure under what justification the authors assert that the approach remains valid. If there is indeed some interaction with the beta transmission terms as the authors suggest, I wonder that they did not try constraining the beta terms in some way as suggested by R1. While fixing beta_seas[13] at 1 reduces the degrees of freedom, perhaps there are identifiability issues between beta_seas and beta_lt that are pushing the model into an unrealistic parameter space.

RESPONSE:

The heterogeneous mixing parameters are indeed lower than we expected (noted in Supplementary Text 2 under Chronic Carriers), which was what led us to further explore model assumptions and formulations in some of the sensitivity analyses. This parameter varied slightly between model formulations, but nevertheless remained low, suggesting that long-cycle transmission of typhoid (due to chronic carriers, *C*, and bacterial contamination of water supplies and the broader environment that is not directly proportional to cases in the previous generation) may help to explain some of the lack of autocorrelation in the data that the short-cycle transmission alone (contaminated food and water in the immediate environment, occurring in direct proportion to cases in the previous generation) does not explain. We have added more explanation to this part of the sensitivity analysis at the end of Supplementary Text 2.

Minor comments:

- I might recommend using a different notation for births since you already have two beta transmission parameters.

RESPONSE:

We have changed the capital “B” to a lowercase “b” so the notation would look less similar to the beta notation. We did not change it to a different letter entirely so we could stay consistent with the common use of the letter “B” or “b” to represent births or birth rates in transmission dynamic models.

- Line 363: I think this should be “S17-S20 figs.” Maybe some of the other supplemental material numberings are off too?

RESPONSE:

This label (S7-S14) is correct; we are referring to the figures showing the plots of both long-term typhoid transmission and the first eight financial predictors (water supply receipts, water supply expenses, sewer system expenses, water supply outlays, sewer system outlays, value of the water supply system, funded debt of the water supply system, and funded debt of the sewer system) and not the two overall investment variables.
